# Temporal relationship between Women’s empowerment and utilization of antenatal care services: lessons from four National Surveys in sub-Saharan Africa

**DOI:** 10.1186/s12884-021-03679-8

**Published:** 2021-03-10

**Authors:** Yusuf Olushola Kareem, Imran Oludare Morhason-Bello, Funmilola M. OlaOlorun, Sanni Yaya

**Affiliations:** 1 Institute for Advanced Medical Research and Training, College of Medicine, University of Ibadan, Ibadan, Nigeria; 2 Department of Obstetrics and Gynaecology, Faculty of Clinical Sciences, College of Medicine, University of Ibadan, Ibadan, Nigeria; 3 Centre for Population and Reproductive Health, College of Medicine, University of Ibadan, Ibadan, Nigeria; 4 Department of Community Medicine, College of Medicine, University of Ibadan, Ibadan, Nigeria; 5 School of International Development and Global Studies, University of Ottawa, 120 University Private, Ottawa, ON K1N 6N5 Canada; 6 The George Institute for Global Health, Imperial College London, London, UK

**Keywords:** Sub-Saharan Africa, Antenatal care, Women’s empowerment, Global Health, Reproductive health

## Abstract

**Background:**

In November 2016, the WHO four-visit focused antenatal care (FANC) model adopted in sub-Saharan Africa (SSA) was reverted to eight contacts or more as a response to reducing the global perinatal and maternal deaths and in achieving the sustainable development goal (SDG) 3. Women’s empowerment, which connote the social standing, position and the ability of women to make life decisions and choices has been associated with the maternal health seeking behaviour and outcomes. This study examined the association between women’s empowerment and the WHO ANC model of eight visits or more, and early first antenatal visit among pregnant women. In addition, we explored the association between women’s empowerment and the WHO FANC model to allow for comparison for countries that have not adopted the recent WHO ANC model.

**Methods:**

The most recent (2018) Demographic and Health Survey (DHS) datasets conducted in SSA were used for analyses. We used all available indicators of women’s empowerment captured in the DHS. The 30 variables on women’s empowerment were classified into eight components using exploratory factor analysis. We fitted separate ordinal logistic regression to assess association between antenatal care utilization (number of visits and time of first antenatal visit) and women empowerment factors while adjusting for other covariates. Analysis was performed with STATA 15.0 and adjusted for complex survey design, *p*-value< 0.05 were used for interpretation of results.

**Results:**

The proportion of women who attended eight or more ANC visits were 1.4, 2.7 and 3.5% in Zambia, Guinea and Mali, respectively. Zambia had the lowest prevalence of 8 or more ANC visits also had the highest prevalence of at least 4 visits (63.8%) and early first ANC visit (38.2%), while Nigeria with the highest prevalence of women with at least 8 visits (17.7%) had the lowest prevalence (17.6%) of women that attended ANC visit in their first trimester. Women’s empowerment was associated with more ANC visits and attending first ANC visit in the first trimester. However, these association with the women empowerment components varied significantly across the four SSA countries.

**Conclusion:**

This study highlights the significant impact of women’s empowerment as a key factor for improving maternal health outcomes in SSA. It is imperative that government and development partners invest more on empowerment of women as part of strategic intervention to improve maternal health outcomes.

**Supplementary Information:**

The online version contains supplementary material available at 10.1186/s12884-021-03679-8.

## Background

Antenatal care (ANC) is an important service delivery to monitor, support and manage pregnant women, the unborn child, and to prepare for childbirth and prevent any complications [1, 2]. It is important that women receive adequate and timely ANC services in order ensure a positive experience during their pregnancy. During ANC, health care providers usually provide health education on warning (danger) signs to avert complications, screen for common infections, offer vaccination or prophylactic treatment to prevent diseases, and counsel for emergencies and birth plan in preparation for childbirth. Generally, ANC utilization has been shown to be an important preventive pillar of maternal morbidity and mortality [1, 3]. Worldwide, 66% (201,000) of an estimated 303,000 maternal deaths experienced in 2015 occurred in sub-Saharan Africa (SSA), making this region to have the highest maternal mortality ratio (MMR) (546/100000 live births), followed by Oceania (187/100000 live births) [4]. Following the Sustainable Development Goal 3 (target 3.1), which is intended to reduce the global MMR to less than 70/100000 live births by the year 2030 [4]. There is a clear evidence that adequate and timely use of ANC services would help reduce the burden of maternal mortality globally, especially in SSA [2].

Due to the high prevalence of preventable pregnancy-related deaths, and in consonance with the SDG target of global reduction in mortality rate, the World Health Organization (WHO) in 2016 released a new guideline and recommendations on ANC care. The new guideline recommended a change from the focused antenatal care (FANC) model of four ANC visits developed in the 1990s to eight contacts or more, due to the new evidences that fewer antenatal visits were associated with more perinatal deaths [1, 3]. The new WHO ANC guideline stipulates that pregnant women should have their first contact in the first 12 weeks of gestation, with subsequent contacts at 20, 26, 30, 34, 38 and 40 weeks of gestation, and also emphasized comprehensive and person-centered care at each contact, provision of timely and relevant information to pregnant women and good conduct among health care seekers and providers [2]. This is to ensure quality and maximum impact of ANC services on the outcome of pregnancy.

Women empowerment is a complex construct that represents social standing, position and the ability of women to make life decisions and choices [5–7]. The decision-making power of a woman is an essential factor that should be considered when ANC utilization is been assessed in any setting, especially, in SSA where women are generally relegated in the society. Empowerment of a woman has been broadly measured using some proxy factors such as economic/financial power, mobility, socio-cultural, labour force participation, attitude towards domestic violence, household decision making, reproductive and sexual decision making [5–13]. Several studies have shown that women’s empowerment is associated with maternal health seeking behavior and outcomes such as utilization of ANC and skilled birth attendant [10, 13–17].

**Fig. 1:**
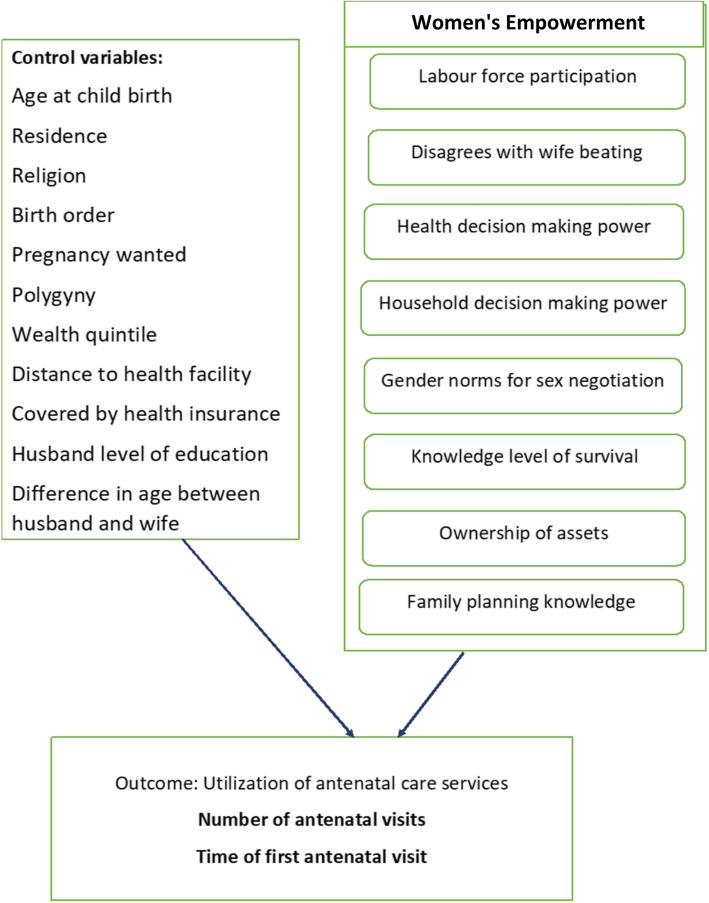
Conceptual Framework of the association between women’s empowerment and utilization of antenatal care services

It is important that the association between women empowerment in SSA and the new WHO ANC protocol utilization is assessed in order to understand real impact across different countries. This study is imperative because the utilization of ANC by women in SSA is a prerequisite to reducing maternal morbidity and mortality, and also, serves as the gateway to accessing other related services including prevention of maternal-to-child transmission of HIV, immunization, family planning and the use of skilled birth attendant for delivery [18]. The study examined the association between women’s empowerment and the 2016 WHO ANC model implementation of eight or more visits and early first antenatal visit among pregnant women. In addition, we explored the association between women’s empowerment and the WHO FANC model to allow for comparison between countries that have not implemented or adopted to the recent WHO ANC visits recommendation (Fig. 1).

## Methods

This study extracted information from woman’s questionnaire in the most recent Demographic and Health Survey (DHS) conducted in SSA. The DHS used a standardized questionnaires, procedures and methodologies comparable across countries to provide up-to-date estimates on several health indicators and also to track a country’s progress towards attaining the SDGs. The DHS sampling technique is usually a two-stage stratified cluster design. The first stage is the selection of clusters, usually called enumeration areas (EAs) within urban and rural strata from the census file which serve as the sampling frame, while the second stage is the selection of households in each of the selected EAs. The survey protocols were reviewed and approved by the ICF Institutional Review Board and the Health Research Ethics Board of the host country to ensure compliance with the county regulation, and also with the generally acceptable good practice of data protection, privacy and confidentiality. The details of sampling methods, procedures and implementation can be found on the DHS website in each country’s final report [19–22]. Ethical approval were obtained from the DHS Program to use secondary data with no identified information traceable to respondents, from their repositories. The dataset for countries where DHS is conducted can be accessed for free after due permission from https://dhsprogram.com .

The inclusion criteria were countries that had data collected more than a year after the November 2016 WHO new guidelines for ANC services was introduced. We explored all datasets available for SSA countries collected in year 2018 or later, taking into account a 9-month gestation period. The eligible countries were Nigeria, Guinea, Mali and Zambia with the most recent surveys conducted and datasets released as at April 2020. The period of data collection and information on sample sizes were presented in (Table 1). To identify factors associated with the WHO recommendations (antenatal visit ≥8 and first antenatal visit ≤3 months), we extracted information on all women with most recent birth within 0 to 12 months prior to the month of interview. Similarly, we conducted a sub analysis to examined the association between women’s empowerment with the existing FANC model (≥4 visits), since not all countries have adopted the ANC model. In the writing of this manuscript, we relied on the Strengthening the Reporting of Observational Studies in Epidemiology (STROBE) guidelines for cross-sectional studies (https://www.equator-network.org/reporting-guidelines/strobe/).

**Table 1:** Country and sample size details

Country	Survey data collection period	Total women (15–49) interviewed	Sample size by design^a^	Selected women sample^b^	% of completed responses
Nigeria	August 14–December 29, 2018	41,821	6857	6709	97.8
Mali	August 6–November 18, 2018	10,519	2049	1937	94.5
Guinea	March 27–June 28, 2018	10,874	1708	1643	96.2
Zambia	July 17–January 24, 2019	13,683	1637	1526	93.2

^a^Sample size by design are women aged 15–49 currently married, who gave birth in the last 0–12 months prior to the month of interview and responded to questions on antenatal care visits and timing ^b^Selected women sample are women with complete response for all variables considered

### Data management

#### Variables and measurements

The outcome variables were number of antenatal visits categorized as < 8 visits or ≥ 8 visits (as well as < 4 visits vs ≥4 visits), and timing of the first antenatal care visit categorized as ≤3 months or > 3 months (as per the WHO recommendations) [2].

The main primary response variable was women’s empowerment; due to complexities in its measurement, we used all available indicators of women’s empowerment captured in the DHS and extracted 30 variables that were classified into 8 components of empowerment measures. These indicators and components had been used in previous studies [5, 8, 9, 11, 13, 15]. In addition, we added variables related to ownership of assets (own a land or house) and social and financial inclusion (own a bank account, own a phone or/and use phone for financial transactions) as measures of women’s empowerment captured in the DHS. Using the exploratory factor analysis, the extracted variables were categorized into 8 components of women’s empowerment. The details of indicators, operationalization and their components were presented in (Additional file 1).

Other independent variables considered to be relevant to ANC utilization included in this analysis were: age of women at childbirth, (categorized as ≤19, 20–24, 25–29, 30–34, 35–39, ≥40), residence (urban vs rural), religion (Christians, Muslims and others), birth order (1–2, 3–4 and ≥ 5), parity (1–2, 3–4 and ≥ 5), pregnancy wanted (no vs yes), polygyny (monogamous, polygamous as first wife and polygamous as second wife or later), distance to health facility (big problem vs not a big problem), covered by health insurance (no vs yes), wealth quintiles (poorest, poorer, middle, richer and richest), husband’s highest educational level (none, primary, secondary and tertiary), difference in age between husband and wife (wife older or same age with husband, husband 1–5 years older, husband 6–10 years older and husband more than 10 years older).

#### Analytical steps and methods

The first step of data analysis was the extraction of information on currently married women that gave birth within a year (0–12 months) prior to the survey, and did not have missing responses on the women empowerment indicators and other covariates considered. The sample sizes ranged from 1526 women in Zambia to 6709 women in Nigeria. A factor analysis was performed based on 30 operationalized variables to extract the components of women empowerment. We investigated the correlation between variables and examined the appropriateness of the use of factor analysis. We conducted the Bartlett’s test of sphericity to show whether there were adequate correlations among items and a Kaiser-Meyer-Olkin (KMO) measure of sampling adequacy. A Bartlett’s test of *p*-value < 0.05 suggest that there are sufficient intercorrelations to conduct the factor analysis, and a larger value of KMO (at least 0.05) is better and provides an overall measure of the overlap or shared variances between pairs of variables, and can uniquely identify items into different factors or components. A quartimin oblique rotation was used to assess for correlations between factors, and thereafter, the solution was then sorted in order to group items into factors. Each component scores were then divided into tertile (low, middle and high). The women empowerment indicators were categorized into eight components by factor analysis. In each country, the Bartlett test of sphericity had a *p*-value< 0.001 and KMO measure of sampling adequacy was greater than 0.05 (0.80 for Mali and Guinea, 0.86 in Zambia and 0.89 in Nigeria), which suggest the appropriateness for the use of factor analysis. The categorization of the women empowerment dimensions were: (1) labour force participation which consist of 5 indicators (2) health decision making power consisting of 3 indicators (3) household decision making power consisting of 4 indicators (4) disagreement with justification to wife beating which consist of 5 indicators (5) gender norms for sex negotiation which consist of 2 indicators (6) family planning knowledge which consist of 3 indicators (7) women’s knowledge level of survival consisting of 6 indicators and (8) ownership of assets consisting of 2 indicators.

The multicollinearity test of the outcome variables and all other covariates were conducted; parity was dropped due to its perfect collinearity with birth order. Only respondents that had complete information and responses on all variables were considered, the percentage responses range from 93% in Zambia to 98% in Nigeria (Table 1). The distribution and prevalence of women with at least 8 ANC visits (as well as those with at least 4 ANC visits) and early timing of first ANC visit (≤3 months of gestation) are presented by the women empowerment dimensions grouping (low, middle and high). Next, we performed a crude ordinal logistic regression between outcome variables and each of the covariates. Finally, an ordinal logistic regression was performed including women’s empowerment variable and adjusting for other covariates. Analysis were performed using STATA 15.0 and adjusted for survey design (weighting, clustering and stratification); while *p*-value< 0.1 were considered for the crude analysis, p-value< 0.05 were used for the interpretation of the adjusted models and results.

## Results

The distribution of women’s characteristics: age at childbirth, birth order, place of residence, wealth quintile, access to health insurance coverage, and difference in age between husband and wife were similar across countries (Table 2). The percentage of adolescent mothers (≤19 years) was lowest in Nigeria (9.1%) and highest in Guinea (13.7%). Most women belong to the poorest and poorer wealth quintiles and mostly reside in rural areas, and less than one in 20 women have access to health insurance coverage. Other variables such as religion, pregnancy wanted, polygyny, distance to health facilities and husband level of education varied across countries. Majority of women were Muslims except in Zambia where 97.5% of the women were Christian and had the least polygyny (10.7%) compared with other countries. The percentage of women whose husbands had no formal education was lowest in Zambia (6.6%) and highest in Mali (74.6%). The percentage of women who attended at least 8 antenatal visits was lowest in Zambia (1.4%) and highest in Nigeria (17.7%). Although, Zambia had the highest proportion of women who made at least 4 ANC visits (63.8%) and also had the lowest proportion of women who do not attend antenatal (1.3%), followed by Guinea (13.0%), Mali (20.9%) and with the highest proportion in Nigeria (24.0%). The percentage of women that started their ANC visit in the first 3 months of gestation was lowest in Nigeria (17.6%) and highest in Zambia (38.2%).

**Table 2:** Descriptive characteristics of currently married women with most recent birth, Nigeria, Guinea, Mali, Zambia DHS 2018 (Weighted)

Variable	Nigeria	Mali	Guinea	Zambia
	Frequency (%)	Frequency (%)	Frequency (%)	Frequency (%)
***Demographic***				
**Number of antenatal visits**				
< 8	5519 (82.3)	1870 (96.5)	1560 (97.3)	1505 (98.6)
≥ 8	1190 (17.7)	67 (3.5)	43 (2.7)	21 (1.4)
**Number of antenatal visits**				
< 4	2983 (44.5)	1084 (56.0)	1071 (65.2)	553 (36.2)
≥ 4	3726 (55.5)	853 (44.0)	572 (34.8)	973 (63.8)
**Time of first antenatal visit**				
≤ 3 months of gestation	1184 (17.6)	687 (35.5)	5525 (26.2)	583 (38.2)
> 3 months of gestation	5525 (82.4)	1250 (64.5)	1183 (73.8)	943 (61.8)
**Age at child birth (years)**				
< 19	610 (9.1)	240 (12.3)	225 (13.7)	164 (10.8)
20–24	1519 (22.6)	489 (25.3)	349 (21.3)	405 (26.6)
25–29	1946 (29.0)	508 (26.2)	444 (27.0)	364 (23.9)
30–34	1393 (20.8)	351 (18.2)	308 (18.8)	299 (19.6)
35–39	841 (12.5)	251 (12.9)	207 (12.6)	204 (13.4)
40–44	398 (6.9)	96 (5.0)	109 (6.7)	212 (8.9)
**Residence**				
Urban	2488 (37.1)	384 (19.8)	414 (25.2)	498 (32.7)
Rural	4221 (62.9)	1553 (80.2)	1229 (74.8)	1028 (67.3)
**Religion**				
Christians	2669 (33.8)	61 (3.1)	155 (9.5)	1488 (97.5)
Muslims	4400 (65.6)	1793 (92.6)	1460 (88.8)	12 (0.8)
Others	40 (0.6)	83 (4.3)	28 (1.7)	26 (1.7)
**Birth order**				
1–2	2361 (35.2)	630 (32.5)	551 (33.5)	577 (37.8)
3–4	1931 (28.8)	567 (29.3)	551 (33.5)	449 (29.4)
≥ 5	2417 (36.0)	740 (38.2)	541 (33.0)	500 (32.8)
**Pregnancy wanted**				
No (later/no more)	759 (11.3)	339 (17.5)	259 (15.8)	541 (35.5)
Yes	5950 (88.7)	1598 (82.5)	1384 (84.2)	985 (64.5)
**Polygyny**				
Monogamous	4692 (69.9)	1282 (66.2)	1007 (61.3)	1362 (89.3)
Polygamous as first wife	742 (11.1)	249 (12.8)	218 (13.3)	69 (4.5)
Polygamous as 2nd **or more**	1275 (19.1)	406 (21.0)	417 (25.4)	95 (6.2)
**Wealth quintiles**				
Poorest	1433 (21.4)	411 (21.2)	443 (26.9)	389 (25.5)
Poorer	1554 (23.2)	422 (21.8)	373 (22.7)	368 (24.1)
Middle	1420 (21.2)	414 (21.4)	325 (19.8)	297 (19.5)
Richer	1239 (18.5)	356 (18.4)	309 (18.8)	219 (14.3)
Richest	1063 (15.8)	333 (17.2)	194 (11.8)	253 (16.6)
**Distance to health facility**				
Big problem	1907 (28.4)	587 (30.3)	838 (51.0)	568 (37.2)
Not a big problem	4802 (71.6)	1350 (69.7)	805 (49.0)	958 (62.8)
**Covered by health insurance**				
No	6574 (98.0)	1840 (95.0)	1625 (98.9)	1497 (98.1)
Yes	135 (2.0)	97 (5.0)	17 (1.1)	28.5 (1.9)
**Husband’s level of education**				
None	2502 (37.3)	1370 (70.8)	1225 (74.6)	100 (6.6)
Primary	861 (12.8)	217 (11.2)	111 (6.78)	569 (37.3)
Secondary	2291 (34.2)	257 (13.3)	199 (12.14)	719 (47.1)
Higher	1056 (15.7)	93 (4.8)	107 (6.5)	138 (9.0)
**Difference in age between husband and wife**				
Wife older or same age	103 (1.5)	34 (1.7)	24 (1.5)	74 (4.8)
Husband 1–5 years older	1614 (24.1)	409 (21.1)	264 (16.1)	829 (54.3)
Husband 6–10 years older	2424 (36.1)	649 (33.5)	514 (31.2)	464 (30.4)
Husband > 10 years older	2568 (38.3)	845 (43.6)	841 (51.2)	160 (10.5)
**Total**	6709	1937	1643	1526

The distribution, prevalence and 95% confidence interval of women that attended at least 8 antenatal visits, and women who had their first ANC visit in the first trimester were presented by women empowerment components Table 3. In the four countries, women were fairly evenly distributed among each tertile of the labour force participation and women’s knowledge level. About two-third of women in Nigeria (66.6%), and less than a third (29.7%) from Mali and Guinea had high disagreement with justification towards wife beating. Almost seven in 10 women in Zambia, and half of women in the other countries had high health decision making power. Three in five women in Mali and Guinea had low household decision making power while two in five of women had low power for sex negotiation. About half of women from Mali, Guinea and Zambia were categorized within the low tertile of ownership of assets, whereas, nearly nine in ten Nigerian women were within this group.

**Table 3:** Distribution and Prevalence of at least 8 ANC visits and early timing of first ANC visit by women’s empowerment components in Nigeria, Mali, Guinea and Zambia DHS 2018

Variable	Nigeria (***N*** = 6709)			Mali (***N*** = 1937)			Guinea (***N*** = 1643)			Zambia (***N*** = 1526)		
	n(%)	visit ≥ 8	≤ 3 months	n(%)	visit ≥ 8	≤ 3 months	n(%)	visit ≥ 8	≤ 3 months	n(%)	visit ≥ 8	≤ 3 months
		Prevalence, % (95% CI)	Prevalence, % (95% CI)		Prevalence, % (95% CI)	Prevalence, % (95% CI)		Prevalence, % (95% CI)	Prevalence, % (95% CI)		Prevalence, % (95% CI)	Prevalence, % (95% CI)
Total (Prevalence, %)		***17.7 (16.4–19.2)***	***17.6 (16.4–19.0)***		***3.5 (2.6–4.6)***	***35.5 (32.8–38.3)***		***2.7 (1.9–3.6)***	***26.2 (23.3–29.4)***		***1.4 (0.9–2.3)***	***38.2 (34.6–41.9)***
**Labour force participation**		p < 0.001	p < 0.001		p = 0.014	p < 0.001		p = 0.095	p = 0.015		*p* = 0.163	p = 0.033
Low	2250 (33.5)	10.4 (8.8–12.3)	12.5 (10.9–14.4)	817 (42.2)	3.6 (2.3–5.6)	36.3 (32.3–40.5)	596 (36.3)	3.1 (2.0–4.9)	25.4 (21.7–29.7)	715 (46.8)	1.0 (0.5–2.1)	38.5 (34.1–43.2)
Middle	2132 (31.8)	14.3 (12.5–16.2)	17.4 (15.5–19.4)	455 (23.5)	1.1 (0.4–3.0)	25.2 (21.2–29.6)	478 (29.1)	1.3 (0.6–2.9)	21.7 (17.4–26.8)	352 (23.1)	2.7 (1.3–5.5)	44.5 (37.3–51.9)
High	2326 (34.7)	28.0 (25.3–30.8)	22.9 (20.5–25.4)	665 (34.3)	4.9 (3.4–7.0)	41.6 (37.4–45.8)	569 (34.6)	3.3 (2.1–5.3)	30.9 (26.2–36.1)	459 (30.1)	1.0 (0.3–3.7)	32.9 (27.2–39.1)
**Disagreement to justification toward wife beating**		p < 0.001	*p* < 0.001		p = 0.008	p = 0.069		*p* = 0.962	p = 0.079		*p* = 0.435	*p* = 0.781
Low	1369 (20.4)	6.3 (4.9–8.2)	10.4 (8.6–12.6)	758 (39.1)	1.8 (1.0–3.2)	31.9 (27.9–36.1)	1608 (37.4)	2.7 (1.5–4.7)	28.2 (23.9–33.0)	381 (24.9)	0.7 (0.2–2.1)	39.8 (33.2–46.6)
Middle	872 (13.0)	10.2 (8.1–12.7)	14.0 (11.5–17.0)	604 (31.2)	5.1 (3.4–7.6)	37.2 (32.7–42.0)	1415 (32.9)	2.8 (1.6–5.1)	21.4 (17.1–26.4)	374 (24.5)	1.9 (0.7–5.0)	38.7 (32.4–45.4)
High	4468 (66.6)	22.7 (20.8–24.7)	20.6 (19.0–22.3)	575 (29.7)	4.0 (2.6–6.1)	38.5 (33.9–41.1)	1281 (29.7)	2.5 (1.5–4.2)	27.8 (23.2–32.9)	772 (50.6)	1.5 (0.7–2.9)	37.2 (33.0–41.7)
**Health decision making power**		p < 0.001	p = 0.001		P < 0.001	p < 0.001		p = 0.045	p = 0.013		*p* = 0.809	*p* = 0.355
Low	1375 (20.5)	14.6 (12.2–17.5)	14.8 (12.5–17.5)	527 (27.2)	0.8 (0.3–2.0)	26.7 (22.3–31.7)	1024 (23.8)	1.7 (0.1–3.2)	21.8 (17.9–26.3)	279 (18.3)	1.4 (0.5–3.9)	42.9 (35.6–50.6)
Middle	1981 (29.5)	13.3 (11.5–15.4)	16.0 (14.1–18.1)	398 (20.6)	2.7 (1.3–5.4)	29.9 (24.9–35.5)	870 (20.2)	2.0 (0.8–4.6)	28.1 (23.8–32.9)	184 (12.0)	2.0 (0.4–9.0)	37.6 (28.3–47.9)
High	3353 (50.0)	21.6 (19.6–23.8)	19.8 (18.0–21.6)	1012 (52.2)	5.1 (3.8–7.0)	42.3 (38.6–46.0)	2409 (56.0)	4.4 (2.9–6.6)	29.9 (25.4–34.9)	1063 (69.7)	1.3 (0.7–2.4)	37.1 (33.4–40.9)
**Household decision making power**		p < 0.001	p < 0.001		*p* = 0.632	p < 0.001		*p* = 0.458	*p* = 0.745		*p* = 0.748	p = 0.001
Low	2672 (39.8)	7.9 (6.7–9.2)	12.0 (10.6–13.6)	1221 (63.0)	3.2 (2.3–4.5)	45.3 (42.0–48.6)	2567 (59.6)	3.3 (2.0–5.4)	26.2 (22.2–30.6)	490 (32.1)	1.8 (0.8–3.9)	46.5 (40.1–53.1)
Middle	1797 (26.8)	15.4 (13.4–17.6)	16.2 (14.2–18.3)	81 (34.2)	2.7 (0.7–10.5)	36.1 (33.2–39.2)	275 (6.4)	2.0 (1.1–3.8)	25.1 (20.7–30.0)	608 (39.9)	1.3 (0.6–3.2)	36.1 (31.4–41.0)
High	2240 (33.4)	31.4 (28.7–34.2)	25.6 (23.2–28.1)	635 (32.8)	4.1 (2.6–6.2)	34.3 (31.1–37.6)	1462 (34.0)	2.5 (1.5–4.3)	27.3 (23.1–32.0)	428 (28.0)	1.1 (0.4–2.7)	31.7 (26.3–37.8)
**Gender norm for sex negotiation**		p < 0.001	p < 0.001		*p* = 0.121	p = 0.020		*p* = 0.233	p = 0.011		*p* = 0.411	*p* = 0.500
Low	2789 (41.6)	8.0 (6.7–9.4)	10.6 (9.3–12.2)	1180 (60.9)	5.3 (2.8–9.9)	32.7 (29.5–36.2)	2507 (58.2)	2.0 (1.2–3.3)	24.2 (21.0–27.8)	356 (23.3)	0.6 (0.1–2.5)	35.6 (28.8–43.0)
Middle	1183 (17.6)	17.0 (14.5–19.9)	20.6 (17.8–23.7)	219 (11.3)	4.4 (2.8–6.9)	41.4 (34.0–49.1)	615 (14.3)	3.1 (1.7–5.5)	23.8 (18.6–30.0)	310 (20.3)	1.8 (0.6–5.5)	36.8 (30.1–44.0)
High	2737 (40.8)	28.0 (25.6–30.6)	23.5 (21.6–25.6)	538 (27.8)	3.5 (2.6–4.6)	39.2 (34.5–44.0)	1183 (27.5)	3.7 (2.1–6.3)	32.9 (27.6–38.6)	860 (56.4)	1.6 (0.9–2.7)	39.8 (35.6–44.1)
**Knowledge level of survival**		p < 0.001	p < 0.001		p < 0.001	p < 0.001		p < 0.001	p < 0.001		*p* = 0.554	*p* = 0.344
Low	2595 (38.7)	3.4 (2.6–4.4)	8.6 (7.3–10.1)	823 (42.5)	0.6 (0.3–1.6)	24.5 (21.2–28.1)	1836 (42.7)	1.1 (0.6–2.1)	20.9 (17.4–25.0)	601 (39.4)	1.3 (0.5–3.1)	40.5 (34.5–46.7)
Middle	1946 (29.0)	14.9 (12.9–17.2)	16.8 (14.7–19.1)	557 (28.8)	3.0 (1.7–5.0)	31.7 (27.7–36.0)	1083 (25.2)	2.0 (1.0–4.3)	28.6 (23.7–34.0)	416 (27.3)	0.9 (0.3–2.6)	38.5 (33.0–44.3)
High	2168 (32.3)	37.4 (34.9–40.1)	29.2 (26.9–31.7)	557 (28.7)	8.1 (5.9–11.2)	55.6 (51.3–59.9)	1385 (32.2)	6.1 (4.1–8.9)	33.0 (27.9–38.6)	508 (33.3)	1.9 (0.9–4.1)	35.3 (30.7–40.2)
**Ownership of assets**		p < 0.001	p < 0.001		*p* = 0.508	*p* = 0.143		p = 0.042	p = 0.001		*p* = 0.700	p = 0.001
Low	5819 (86.7)	16.9 (15.5–18.5)	16.8 (15.5–18.2)	1039 (53.6)	3.9 (2.7–5.6)	30.9 (24.8–37.8)	2360 (54.8)	3.4 (2.3–5.0)	29.3 (25.5–33.5)	670 (43.9)	1.4 (0.8–2.6)	32.1 (27.9–36.5)
Middle	132 (2.0)	30.3 (22.5–39.3)	33.8 (25.6–43.2)	290 (15.0)	2.4 (1.2–5.1)	34.0 (29.4–38.8)	684 (15.9)	2.6 (1.5–4.6)	25.6 (21.3–30.4)	673 (44.1)	1.2 (0.5–2.8)	42.9 (37.3–48.6)
High	758 (11.3)	21.9 (18.5–25.6)	21.5 (18.4–24.9)	607 (31.4)	3.2 (2.0–5.2)	35.5 (32.8–38.3)	1260 (29.3)	0.6 (0.2–2.6)	18.1 (13.9–23.1)	183 (12.0)	2.2 (0.5–8.8)	43.5 (35.4–52.0)
**Family planning knowledge**		p < 0.001	p < 0.001		*p* = 0.190	*p* = 0.565		p = 0.012	p < 0.001		*p* = 0.913	*p* = 0.164
Low	3640 (54.3)	11.7 (10.4–13.2)	13.9 (12.5–15.4)	834 (43.0)	2.6 (1.6–4.1)	37.6 (33.8–41.6)	531 (31.3)	1.8 (0.9–3.7)	19.6 (16.1–23.5)	400 (26.2)	1.2 (0.5–3.1)	39.7 (33.5–46.3)
Middle	836 (12.5)	15.1 (12.3–18.4)	17.1 (14.4–20.2)	821 (42.4)	4.5 (3.0–6.6)	38.9 (36.5–41.4)	740 (45.0)	2.1 (1.3–3.4)	27.8 (23.6–32.5)	883 (57.9)	1.4 (0.7–2.7)	36.1 (31.7–40.7)
High	2233 (33.3)	28.6 (25.8–31.6)	24.0 (21.8–26.4)	282 (14.6)	3.2 (1.6–6.6)	38.6 (36.5–40.7)	372 (22.7)	4.9 (3.1–7.8)	32.6 (27.1–38.6)	243 (15.9)	1.7 (0.7–4.1)	43.6 (36.9–50.6)

More than half of Nigerian women (54.3%) and about a third of women from Guinea (31.3%) had low knowledge of family planning. The prevalence of women that attended at least 8 ANC visits ranged from 1.4% (95% CI: 0.9–2.3%) in Zambia to 17.7% (95% CI: 16.4–19.2%) in Nigeria. Also, Zambia had the highest prevalence of women with at least 4 ANC visit (*p* = 63.8%; 95% CI: 60.3–67.1%)) while Guinea (*p* = 34.8%; 95% CI: 31.5–38.3%) had the lowest (Additional file 2). The proportion of women that registered early for their first antenatal visits was lowest in Nigeria 17.6% (95% CI: 16.4–19.0%) in Nigeria and to 38.2% (95% CI: 34.6–41.9%) in Zambia.

The association between prevalence of at least 8 ANC visits and women’s empowerment variable varied across the four countries. Specifically, labour force participation was significantly associated with ANC visits in Nigeria (*p* < 0.001), Mali (*p* = 0.014) and Guinea (*p* = 0.095) only. Although the relationship was linear in Nigeria, however, the prevalence of ANC visits was higher at the extreme of labour force participation category relative to the middle group in Mali and Guinea. Disagreement towards wife beating was associated with ANC visit in Nigeria (*p* < 0.001) and Mali (*p* = 0.008). There was a linear significant association between women health decision making power and ANC attendance in Nigeria (*p* < 0.001), Mali (*p* < 0.001) and Guinea (*p* = 0.045), a similar association was also observed with women’s knowledge about survival in the three countries (*p* < 0.001). However, only in Nigeria (*p* < 0.001) was household decision and gender norms for sex negotiation was associated with ANC visit. Although, ownership of assets was associated with ANC visits in Nigeria (*p* < 0.001) and Guinea (*p* = 0.042), but the relationship was positive in Nigeria and inverse in Guinea. Family knowledge had a linear association with ANC visits in Nigeria (*p* < 0.001) and Guinea (*p* = 0.012). Similar patterns of association were observed using the FANC model of at least 4 visits.

Women’s empowerment and the first ANC visit within 3 months of conception also varied across the four countries. In brief, labour force participation was significantly associated with having first ANC visit in the first trimester in the four countries (Nigeria and Mali, *p* < 0.001; Guinea, *p* = 0.015; Zambia, *p* = 0.033) with only Nigeria showing a linear trend. The disagreement towards wife beating (Nigeria, *p* < 0.001; Mali, *p* = 0.069; Guinea, *p* = 0.079), health decision making power of women (Nigeria, *p* = 0.001; Mali, *p* < 0.001; Guinea, *p* = 0.013), gender norm for sex negotiation (Nigeria, *p* < 0.001; Mali, *p* = 0.020; Guinea, *p* = 0.011) and women’s knowledge of survival (all four countries, *p* < 0.001) factors were associated with history of first ANC visit during the first trimester among women in Nigeria, Mali and Guinea. The report of first ANC visit in the first trimester was associated with household decision making power in Nigeria (*p* < 0.001), Mali (*p* < 0.001) and Zambia (*p* = 0.001), ownership of assets in Nigeria (*p* < 0.001), Guinea (p = 0.001) and Zambia (*p* = 0.001) and knowledge about family planning in Nigeria (*p* < 0.001) and Guinea (*p* < 0.001).

### Multivariable ordinal logistic regression between women empowerment factors and number of ANC visits

In Nigeria, the crude analysis showed that all women empowerment factors were positively associated with higher odds of reporting at least 4 and 8 ANC visits, except that women in the middle tertile of health decision making were not significantly associated with attending 8 or more visits (Additional file 3, Additional file 4). Similarly, all Other covariates positively associated with at least 4 and 8 ANC visits were wealth quintiles, report of no big problem to health facility, access to health insurance coverage, husband’s level of education and women aged between 20 and 39 years at last childbirth compared to adolescent mothers. However, women in rural areas, Muslims and other faith organizations besides Christians, history of high birth order, report of pregnancy been wanted and those in polygamous union were less likely to attend at least 4 or 8 ANC visits.

In Mali, disagreement with wife beating, health decision making power, gender norm for sex negotiation, women’s knowledge of survival and knowledge about family planning were positively associated with higher odds of reporting at least 4 and 8 ANC visits relative to others that reported less than 4 and 8 visits. However, only women categorized to be in the middle tertile of labour force participation in Mali had lower odds of reporting at least 4 and 8 ANC visits than others. Women in rural areas and birth order had a lower odds of at least 4 and 8 ANC visits, only women with birth order of the last pregnancy greater than five had lower odds to report at least 8 ANC visits, while women in the richest wealth quintiles, who had no big problem to reach health facility, covered by health insurance and whose husband had higher level of education were positively associated with the report of at least 4 and 8 ANC visits.

In Guinea, household decision making power, women’s knowledge of survival and knowledge about family planning were positively associated with higher odds of having at least 4 and 8 ANC visits. Conversely, women in the high tertile of asset ownership had lower odds of attending at least 4 or 8 ANC visits and those in the middle tertile of labour force participation had lower odds of reporting at least 8 ANC visits. Women that reported that the index pregnancy was wanted, from a richer or richest wealth quintiles, who did not report a big problem to reach a health facility and whose husband had post-secondary education were positively associated with high odds of reporting at least 8 ANC visit. On the contrary, women living in rural areas and with birth order of the last pregnancy greater than four had lower odds of reporting at least 4 and 8 ANC visit.

Of all the women empowerment factors, only women categorized to be in the middle tertile of labour force participation had higher odds of attending at least 4 and 8 ANC visits in Zambia. Gender norm for sex negotiation, ownership of assets and high knowledge about family planning were associated with higher odds of attending at least 4 ANC visits. Women aged 30–34 years, covered by health insurance, and whose husband had secondary or post-secondary education, and with birth order of the last pregnancy greater than two had lower odds of reporting at least 8 ANC visits.

In the adjusted model, there were higher odds of reporting attendance of at least 8 ANC visits among Nigerian women categorized into high tertiles of some women empowerment factors such as labour force participation (Adjusted odds ratio [AOR] = 1.81, 95%CI 1.41–2.32), household decision making power (AOR = 1.53, 95%CI 1.19–1.96), knowledge for survival (AOR = 2.40, 95%CI 1.68–3.45), gender norm for sex negotiation (AOR = 1.35, 95%CI 1.07–1.70), and knowledge of family planning (AOR = 1.26, 95%CI 1.02–1.56) relative to those in the low tertiles. Furthermore, women in Nigeria classified in the middle tertile of household decision making power and women’s knowledge level of survival were associated with 1.38 (95%CI 1.09–1.73) and 1.92 (95%CI 1.39–2.26) odds, respectively, compared to those in the low tertile to report 8 or more ANC visits. Similarly, labour force participation, knowledge level of survival, high disagreement to wife beating (AOR = 1.27, 95%CI 1.03–1.56), high health decision making power (AOR = 1.31, 95%CI 1.05–1.64), high household decision making power (AOR = 1.31, 95%CI 1.07–1.60) and high knowledge about family planning (AOR = 1.52, 95%CI 1.30–1.78) were associated with a higher odds of attending at least 4 ANC visits.

There exist a direct positive association between women aged 25 years and above at last childbirth and those from middle wealth quintiles and above, with the highest odds among women aged 40 years and above (AOR = 2.26, 95%CI 1.17–4.39) and from highest wealth quintiles (AOR = 2.43, 95%CI 1.46–4.04. Women whose husband education was primary (AOR = 1.71, 95%CI 1.15–2.54) and secondary (AOR = 1.51, 95%CI 1.03–2.22) were positively associated with history of at least 8 ANC visits relative to women with husband without formal education. However, women residing in rural areas, who were Muslims, that did not have a big problem to get to health facility and had a birth order of 3–4 or 5 and above were associated with lower odds of reporting ANC visits of at least 8 or more.

In Mali, women in the middle tertile of health decision making power, gender norms for sex negotiation and high ownership of assets; and high and middle tertile of knowledge level of survival were associated with the odds of 4.59, 2.74, 2.13, 4.78 and 3.84, respectively, to attend at least 8 or more ANC visits. However, women with the birth order of 5 and above, and whose husbands were at least 10 years older were associated with the odds of 0.26 and 0.23, respectively, to attend 8 or more ANC visits. While women’s knowledge level of survival and those categorized as high ownership of asset (AOR = 1.49, 95%CI 1.14–1.96) have a higher odds of reporting more than 4 ANC visits.

The only factor associated with the attendance of 8 or more ANC visits among Guinean women was the higher odds of reporting no big problem to access health facility (AOR = 4.02, 95%CI 1.39–11.65). While household decision making power, gender norms for sex negotiation, knowledge about family planning were associated with higher odds of at least 4 ANC visit. Similarly, women categorized to be in the high tertile of labour force participation (AOR = 1.65, 95%CI 1.23–2.23) were associated with a higher odds while those with high disagreement to wife beating (AOR = 0.67, 95%CI 0.49–0.92) had a lower odds of reporting 4 or more ANC visits. In Zambia, women in the middle tertile of labour force participation (AOR = 6.75, 95%CI 2.55–17.86), that had health insurance coverage (AOR = 11.4, 95%CI 1.92–67.55) and whose husbands had post-secondary education were associated higher odds of reporting 8 or more ANC visits. Also, gender norms for sex negotiation power and women in the high tertile of ownership of assets (AOR = 1.61, 95%CI 1.06–2.45) were associated with higher odds of reporting at least 4 ANC visits. However, women with the current birth order of three to four (AOR = 0.05, 95%CI 0.10–0.27), five and above (AOR = 0.01, 95%CI 0.02–0.06), increasing birth order were associated with lower odds of attending 8 or more ANC visits.

#### Women’s empowerment and early timing of first ANC visit

In the unadjusted model, in Nigeria, all women empowerment components were positively associated with the report of ANC visit in the first trimester in Nigeria (Additional file 5). In addition, women in the ages of 20–34 years at last childbirth, wealth quintiles, who had no big problem to get to health facility and husband education were factors associated with the higher odds to attend the first ANC visit in the first trimester early. However, women residing in rural areas, that were Muslims, and who had higher birth order than one and in a polygamous union had a lower odds of attending the first ANC visit in the first trimester. In Mali, gender norms for sex negotiation, knowledge level of survival, women with high disagreement with justification to wife beating, health decision making power, household decision making power and medium labour force participation were positively associated with first ANC visit. In addition, women whose pregnancies were wanted, in the middle to richest quintiles, covered by insurance and husband had post primary education had higher odds of attending their first ANC visit early. Women aged 30 years and above, that were residing in rural areas, in a polygamous union as first wife and whose husband was 1–10 years older were associated with the lower odds to attend the first ANC in the first trimester.

The health decision making, knowledge level of survival, family planning, high labour force participation, gender norms for sex negotiation were positively associated with the higher odds to attend the first ANC visit early among women in Guinea. Similarly, Guinean women with high wealth quintiles, whose age was 30–34 years, of Christian faith, had no big problem to get to health facility, and whose husbands had post-primary education had higher odds of attending first ANC visit in the first trimester. While women with high ownership of asset, in middle tertile of disagreement with justification to wife beating and residing in rural areas were associated with the lower odds to attend first ANC visit early. In Zambia, the factors such as ownership of asset, living in rural area and having husband whose age was more than 10 years were associated with the higher odds of first ANC visits. However, the household decision making, having an age between 35 and 39 years, high birth order and wealth quintiles were associated with the lower odds to attend the first ANC visit in the first trimester.

In Nigeria, the adjusted model showed that labour force participation, gender norms for sex negotiation, women with high knowledge for survival (AOR = 1.40, 95%CI 1.01–1.92), family planning (AOR = 1.24, 95%CI 1.04–1.47) and middle tertile of ownership of assets (AOR = 1.68, 95%CI 1.14–2.48) were positively associated with early timing of first antenatal visit in Nigeria. Women that said their pregnancy were wanted (AOR = 1.70, 95%CI 1.29–2.24), high wealth quintiles, and those whose husband had primary or secondary education had higher odds relative to others without these respective groups. However, Muslim women (AOR = 0.69, 95%CI 0.53–0.88) and those with 3 or more birth order were less likely to attend first antenatal visit in the first 3 months of gestation.

In Mali, women in high tertile of knowledge level of survival (AOR = 1.75, 95%CI 1.27–2.41), ownership of assets (AOR = 1.31, 95%CI 1.01–1.70), in middle tertile of gender norms for sexual negotiation (AOR = 1.54, 95%CI 1.04–2.30) were positively associated with having the first ANC visit in the first trimester. Whereas, women aged 35-39 years (AOR = 1.89, 95%CI 1.04–3.43), living in rural areas (AOR = 1.64, 95%CI 1.10–2.44), described their pregnancies has been wanted (AOR = 1.78, 95%CI 1.31–2.44), and from richer (AOR = 1.85, 95%CI 1.17–2.93) and richest wealth quintiles (AOR = 3.45, 95%CI 1.79–6.63) were associated with higher odds of reporting early first ANC visit in the first 3 months of gestation. However, women with birth order of 3 to 4 (AOR = 0.71, 95%CI 0.51–1.00), and 5 or more (AOR = 0.59, 95%CI 0.38–0.92), and women whose husband were 1 to 5 years (AOR = 0.45, 95%CI 0.21–0.97) older had a lower odds of attending the first ANC visit in their first trimester. In Guinea, women classified in the middle (AOR = 1.54, 95%CI 1.10–2.16) and high tertile of family planning knowledge (AOR = 1.52, 95%CI 1.04–2.20), Muslim women (AOR = 4.46, 95%CI 2.16–9.23), higher wealth quintiles and husband with post-secondary education (AOR = 1.97, 95%CI 1.18–3.27) were positively associated with report of visiting ANC visit in the first trimester. However, women in the middle tertile of disagreement with justification for wife beating had lower odds (AOR = 0.71; 95% CI: 0.50–0.99) to attend first ANC visit in the first trimester. In Zambia, household decision making power, ownership of asset, residing in rural areas (AOR = 1.84, 95%CI 1.20–2.84), and whose husband were 10 years older or more (AOR = 2.43, 95%CI 1.24–4.78) were positively associated with early first ANC visit, while women with 5 or more birth order (AOR = 0.56, 95%CI 0.34–0.93) had a lower odds of attending their first ANC visit before or at 3 months of gestation.

## Discussion

The study showed that women’s empowerment was generally associated with the number of ANC visit and timing of the first ANC visit with variation in the empowerment dimensions among the four countries. While knowledge level of survival was associated with at least 4 and 8 ANC visits in Nigeria and Mali, some women empowerment factors such as labour force participation, family planning, household decision making were associated with reporting more than 4 or 8 ANC visits in Nigeria. Similarly, disagreement with justification to wife beating and health decision making power were associated with attending more than 4 visits while gender norms for sex negotiation were positively associated with reporting more than 8 ANC visit in Nigeria. In addition, ownership of asset was associated with reporting more than 4 or 8 ANC visits and health decision making power, gender norm for sexual negotiation were associated with attending 8 or more visits.

Only labour force participation was positively associated with at least 8 ANC visits in Zambia while gender norm for sex negotiation and family planning were associated with reporting 4 or more visits. None of the women empowerment components were associated with ANC visit in Guinea. Meanwhile, household decision making power, gender norm for sex negotiation, family planning, labour force participation and disagreement with justification to wife beating were associated with attending at least 4 ANC visits.

Furthermore, labour force participation, disagreement to justification towards wife beating, health decision making power and knowledge level for survival were positively associated with timing of first ANC visit in the first trimester among Nigerian and Malian women. Only family planning in Guinea, household wealth and ownership of assets in Zambia showed positive association with early first ANC visit.

In our study, the cut-off was based on the recent WHO guideline of at least eight ANC visits. In addition, this study provided the most recent information on ANC utilization after the introduction of the new WHO recommendation on ANC visits in SSA, More so, our study is also comparable with previous studies that were based on the FANC model of at least four ANC visits [10, 15, 23, 24]. Generally, this study corroborated other findings that showed positive association between different components of women empowerment and utilization of antenatal services [10, 14–16].

This study showed that Nigerian women had the highest proportion (18.0%) of those that utilized at least 8 ANC services, followed by Mali (3.5%), Guinea (2.7%) and Zambia (1.4%). Unexpectedly, Zambia that had the lowest prevalence of 8 ANC visits had the highest prevalence (38.2%) for early first ANC visit while Nigeria with the highest prevalence of women with at least 8 visits has the lowest prevalence of women who attended ANC visit in their first trimester. Although, the prevalence of women who attended at least 4 ANC visits was highest in Zambia (63.8%). The prevalence of first ANC visit in the first trimester in Nigeria (18%) and Zambia (38.2%) were similar to the overall prevalence of first ANC visit in the first trimester in the five-year coverage of NDHS reports in both countries.

Although, the findings from some studies had shown that early ANC visit may not be related to adequate utilization of ANC services. The poor utilization might be related to the negative attitude of healthcare providers and not due to the timing of the first ANC visit [25, 26]. There are studies from Nigeria that have also found that early ANC visit might not translate to high number of visits and likelihood of having a supervised childbirth delivery [27, 28]. Another study in Ghana showed that some health workers sometimes dissuade pregnant women from hospital supervised delivery [29]. Although, a study from Nigeria reported that negative attitudes of health care providers towards ANC attendees might be due to stress from shortage of manpower, poor record keeping and uncompromising attitude of some patients [30]. Despite these challenges, there is evidence that in most SSA countries that ANC utilization might be improving. For example, ANC coverage increased from 58% in 2008 to 67% in 2018 while supervised childbirth by skilled birth attendants increased from 39% in 2008 to 43% in 2018 in Nigeria [20]. The same pattern of increasing ANC coverage and supervised vaginal delivery by healthcare provider had been reported in Kenya, Zambia, Senegal, Uganda and Cameroun [31, 32].

Although women empowerment has been consistently shown to be associated with positive maternal health outcomes, but the composite factors that were considered to define empowerment were different. In this study, it was shown that all measures of women empowerment were associated with the utilization of ANC services, especially, in Nigeria and Mali. However, the relationship between women empowerment components in Guinea was mixed while only ownership of asset and labour force participation among other empowerment components were found to be positively associated with first ANC visit and the recommended number of ANC visit, respectively, in Zambia. Several studies from many SSA Africa had also shown that other proxy factors of empowerment such as western education, high socio-economic class and gainful employment are more likely to utilize ANC and hospital supervised delivery services [10, 32].

This study further strengthened the growing evidence that women empowerment measures influence the antenatal care utilization in SSA. Some studies in SSA [10, 12] found weak association between women autonomy and the use of maternal healthcare services. It is also evident from this study that planned pregnancy, partner education and household wealth showed positive association with ANC utilization while high birth order had a negative association with antenatal care services. It is imperative that policy makers should focus more on women that had no attributes of women empowerment measures and other related factors in the drive to improve access to maternal health services in SSA.

### Strengths and limitations

This study has some potential limitations, the cross-sectional nature of DHS dataset do not allow for causality in interpretation. Similarly, this study used all the indicators for women empowerment in the DHS and this limited our sampled population to currently married women only. Also, in this study we did not adjust for quality of ANC and previous pregnancy care experience from healthcare provider, which may influence decisions to attend or use ANC services [25, 26]. .Despite these limitations, the study has a number of strengths. The analysis was based on the most recent nationally representative datasets of women of reproductive age from four countries in SSA. The study used two key indicators of ANC utilization – early visit to ANC and number of visits. To our knowledge, this study is the first to report association between women empowerment and the new WHO recommendation of eight or more antenatal visits. Similarly, we also analyzed and compared our results with the FANC model of at least 4 visits since some of these countries may not have adopted the new WHO ANC care recommendation of 8 or more contacts. Furthermore, we expanded the measure of women empowerment to include eight components (labour force participation, disagreement with justification towards wife beating, health decision making, household decision making, gender norms for sex negotiation, knowledge level, ownership of asset and family planning).

## Conclusion

The study further emphasized the impact of women’s empowerment on maternal health outcomes and highlighted the roles of the empowerment variables in Nigeria, Mali, Guinea and Zambia, as proxy for other countries in SSA. The appropriate timing, frequency and adequacy of antenatal care visit is necessary to ensure feto-maternal health and prevent pregnancy related complications.

Despite the WHO ANC care recommendation which is geared to reduce mortality related to pregnancy, most of the countries in SSA may not have implemented this recommendation to increase minimum of ANC visits from four to eight contacts, this is important to ensure reduction in childbirth complications and to reduce the high burden of maternal and neonatal mortality rates in the SSA region. It is important that pregnant women should visit health care provider early and comply with the new WHO recommendation of one contact in the first trimester, two contacts in the second trimester (at 20 and 26 weeks of gestation) and five contacts in the third trimester (at 30, 34, 36, 38 and 40 weeks). More importantly, policy aimed at quality antenatal service and promoting good interaction between health care seekers and health care providers whether in government or private parastatals should be integrated in the health system. In addition, future studies should explore reasons for the non implementation of the new WHO ANC model in many SSA settings, and also to compare the quality of care among pregnant women who have adhered to the new ANC care model and those who still adopt the FANC model.

## Supplementary Information

**Additional file 1.** Variables extracted for the composition of women empowerment Indicators.

**Additional file 2.** Distribution and Prevalence of at least 4 ANC visits by women empowerment components in Nigeria, Mali, Guinea and Zambia DHS 2018.

**Additional file 3.** Unadjusted and Adjusted ordinal logistic regression of the association between background characteristics and ≥ 8 ANC visits in Nigeria, Mali, Guinea and Zambia DHS 2018.

**Additional file 4.** Unadjusted and Adjusted ordinal logistic regression of the association between background characteristics and ≥ 4 ANC visits in Nigeria, Mali, Guinea and Zambia DHS 2018.

**Additional file 5.** Unadjusted and Adjusted ordinal logistic regression of the association between background characteristics and early timing of ANC visits (≤ 3 months gestation) in Nigeria, Mali, Guinea and Zambia DHS 2018.

## Data Availability

This study used a secondary dataset from Measure DHS program, the dataset can be accessed after due permission from the DHS program archive and can be downloaded at https://dhsprogram.com/data/available-datasets.cfm.

## Abbreviations

ANC: Antenatal care
DHS: Demographic health survey
EAs: Enumeration areas
FANC: Focused antenatal care
KMO: Kaiser-Meyer-Olkin
SDG: Sustainable development goal
SSA: Sub-Saharan Africa
WHO: World Health Organization
