# Supplementary material for: Temporal relationship between Women’s empowerment and utilization of antenatal care services: lessons from four National Surveys in sub-Saharan Africa
Source: BMC Pregnancy Childbirth. 2021 Mar 10;21:198. doi: 10.1186/s12884-021-03679-8 (PMC7944901; doi:10.1186/s12884-021-03679-8)
Supplement: Supplementary file 1 — Additional file 1. Variables extracted for the composition of women empowerment Indicators. [file 12884_2021_3679_MOESM1_ESM.docx]

Supplementary Table 1. Variables extracted for the composition of women empowerment Indicators

| Dimension (variables) | Operationalization (recoded scores for factor analysis) |
| --- | --- |
| **1.Labour force participation^a^**  Women worked in the last 12 months  Who does woman work for?  Type of earnings from woman's work  Type of occupation  Work all years | No=0; Yes=1  Not working=0; work for family=1, someone else=2 self-employed=3  Not working/Not paid=0; kind alone=1, cash and kind=2; cash only=3  None=0; unskilled=1; others (skilled, clerical, service) =2; professional=3  Not working=0; occasional/seasonal/temporary=1; all year=2 |
| **2.Health decision making power**  Permission to get medical help  Getting money for treatment  Not wanting to go alone to health centres | Big problem=0; not a big problem=1  Big problem=0; not a big problem=1  Big problem=0; not a big problem=1 |
| **3.Household decision making^b^**  Who decides on respondent health care?  Who decides on large household purchases?  Who decides on visit to family or relatives?  Who usually decides on what to do with husband earnings? | Others=0; Jointly with spouse=1, Alone=2  Others=0; Jointly with spouse=1, Alone=2  Others=0; Jointly with spouse=1, Alone=2  Others=0; Jointly with spouse=1, Alone=2 |
| **4.Disagrees wife beating justified**  **G**oes out without telling husband  Neglects the children  Argue with husband  Refuses to have sex with husband  Burns food | Yes=0; No=1  Yes=0; No=1  Yes=0; No=1  Yes=0; No=1  Yes=0; No=1 |
| **5.Gender norms for sex negotiation**  Can ask partner to use condom  Can refuse sex | No=0; Yes=1  No=0; Yes=1 |
| **6.Family planning knowledge**  Unmet need  Heard FP on radio, tv, print media or text message  Knowledge about contraceptive methods | Others (unmet need for spacing and/or limiting) =0; No unmet need (spacing and/or limiting) =1  Not heard about FP from all sources=0; heard from 1 or 2 sources=1; heard from 3 or all sources=2  No method=0; folkloric/traditional=1; modern=2 |
| **7.Women’s knowledge level of survival**  literacy  Highest level of education  Exposure to mass media (newspaper, tv and radio)  Use of internet  Have a bank account  Own a phone or/and for transaction | Cannot read/No card/visually impaired=0; read only part=1; read whole=2  None=0; primary=1; secondary=2, tertiary=3  Not at all=0; access to some media=1, access to all types of media=2  Never=0; yes, in the last 12 months=1; yes, before last 12 months=2  No=0; Yes=1  Do not own a phone=0; own a phone=1; own a phone for transaction=2 |
| **8.Ownership of assets**  Own a land  Own a house | Does not own=0; jointly only=1; alone only=2; jointly and alone=3  Does not own=0; jointly only=1; alone only=2; jointly and alone=3 |

*^a^Earn more than husband were excluded from labour force participation, because only women who have worked in the last 12 months were asked, ^b^Who usually decides on how to spend women’s earning were excluded from household decision because only women who have worked in the last 12 months were asked*
