# Supplementary material for: Temporal relationship between Women’s empowerment and utilization of antenatal care services: lessons from four National Surveys in sub-Saharan Africa
Source: BMC Pregnancy Childbirth. 2021 Mar 10;21:198. doi: 10.1186/s12884-021-03679-8 (PMC7944901; doi:10.1186/s12884-021-03679-8)
Supplement: Supplementary file 2 — Additional file 2. Distribution and Prevalence of at least 4 ANC visits by women empowerment components in Nigeria, Mali, Guinea and Zambia DHS 2018. [file 12884_2021_3679_MOESM2_ESM.docx]

Supplementary Table 2. Distribution and Prevalence of at least 4 ANC visits by women empowerment components in Nigeria, Mali, Guinea and Zambia DHS 2018

| Variable | Nigeria (N=6709) | Mali (N=1937) | Guinea (N=1643) | Zambia (N=1526) |
| --- | --- | --- | --- | --- |
|  | Prevalence,% (95% CI) | Prevalence,% (95% CI) | Prevalence,% (95% CI) | Prevalence,% (95% CI) |
| **Total (Prevalence,%)** | 55.5(53.5-57.6) | 44.0(41.0-47.2) | 34.8(31.5-38.3) | 63.8(60.3-67.1) |
| **Labour force participation** | p<0.001 | p<0.001 | p<0.001 | p=0.162 |
| Low | 44.9(41.8-48.0) | 44.2(39.9-48.6) | 30.2(26.1-34.6) | 62.3(58.0-66.5) |
| Middle | 53.5(50.8-56.3) | 33.4(28.5-38.7) | 26.2(21.3-31.7) | 69.1(62.8-74.8) |
| High | 67.7(64.7-70.6) | 51.1(46.1-56.1) | 46.9(41.9-52.1) | 61.9(55.4-68.0) |
| **Disagreement with justification to wife beating** | p<0.001 | p=0.054 | p=0.378 | p=0.545 |
| Low | 38.5(34.4-42.8) | 40.1(35.7-44.7) | 36.7 (31.6-42.1) | 61.2(54.3-67.7) |
| Middle | 47.3(43.4-51.4) | 46.3(41.6-51.1) | 35.5(29.9-41.6) | 63.0(55.9-69.6) |
| High | 62.4(59.7-64.9) | 46.8(41.9-51.8) | 32.0(27.4-37.0) | 65.4(61.2-69.4) |
| **Health decision making power** | p<0.001 | p<0.001 | p<0.001 | p=0.851 |
| Low | 40.5(36.7-44.4) | 31.8(27.1-36.9) | 27.5(23.0-32.5) | 62.1(54.6-69.1) |
| Middle | 52.6(49.6-55.5) | 40.1(34.1-46.4) | 34.5(29.3-40.0) | 64.8(56.5-72.3) |
| Highest | 63.5(60.7-66.1) | 52.0(48.0-55.9) | 44.1(38.8-49.5) | 64.0(60.1-67.7) |
| **Household decision making power** | p<0.001 | p<0.001 | p<0.001 | p=0.297 |
| Low | 43.2(40.2-46.2) | 39.7(36.2-43.2) | 27.0(22.8-31.7) | 66.8(61.5-71.6) |
| Middle | 53.5(50.5-56.5) | 52.7(39.2-65.8) | 35.5(30.5-40.8) | 63.3(58.6-67.8) |
| High | 72.0(69.4-74.4) | 51.4(46.3-56.4) | 42.0(37.3-46.8) | 61.0(54.6-67.0) |
| **Gender norm for sex negotiation** | p<0.001 | p=0.046 | p<0.001 | p<0.001 |
| Low | 42.1(39.3-44.9) | 41.3(37.8-45.0) | 27.6(23.8-31.6) | 52.3(46.1-58.5) |
| Middle | 58.0(54.2-61.7) | 47.1(39.6-54.8) | 37.4(30.9-44.5) | 70.9(63.8-77.2) |
| High | 68.2(65.6-70.7) | 48.8(43.0-54.6) | 48.9(43.2-54.7) | 65.9(61.8-69.9) |
| **knowledge level of survival** | p<0.001 | p<0.001 | p<0.001 | p=0.687 |
| Low | 31.7(28.9-34.5) | 28.7(25.0-32.7) | 25.9(22.0-30.2) | 62.1(56.6-67.3) |
| Middle | 56.3(53.1-59.4) | 43.0(38.3-47.8) | 65.2(59.5-70.4) | 65.1(58.7-71.0) |
| High | 83.5(81.7-85.1) | 67.8(63.1-72.2) | 49.3(43.9-54.8) | 64.7(59.5-69.5) |
| **Ownership of assets** | p<0.001 | p=0.097 | p=0.047 | p=0.012 |
| Low | 54.2(52.0-56.5) | 46.6(42.6-50.6) | 37.5(33.1-42.1) | 58.5(54.0-62.8) |
| Middle | 72.5(63.0-80.4) | 38.1(31.5-45.1) | 34.0(28.7-39.8) | 67.3(61.6-72.6) |
| High | 62.6(58.5-66.5) | 42.5(37.3-48.0) | 28.2(22.6-34.6) | 70.0(61.5-77.3) |
| **Family planning knowledge** | p<0.001 | p=0.062 | p<0.001 | p=0.071 |
| Low | 45.9(43.3-48.4) | 40.3(36.1-44.6) | 25.0(20.7-29.9) | 0.63(0.57-0.69) |
| Middle | 52.2(48.2-56.2) | 47.2(42.8-51.6) | 36.0(31.3-40.9) | 0.62(0.57-0.66) |
| High | 72.6(70.0-75.0) | 45.9(38.6-53.4) | 46.5(40.5-52.6) | 0.72(0.65-0.78) |
