# Supplementary material for: Temporal relationship between Women’s empowerment and utilization of antenatal care services: lessons from four National Surveys in sub-Saharan Africa
Source: BMC Pregnancy Childbirth. 2021 Mar 10;21:198. doi: 10.1186/s12884-021-03679-8 (PMC7944901; doi:10.1186/s12884-021-03679-8)
Supplement: Supplementary file 3 — Additional file 3. Unadjusted and Adjusted ordinal logistic regression of the association between background characteristics and ≥ 8 ANC visits in Nigeria, Mali, Guinea and Zambia DHS 2018. [file 12884_2021_3679_MOESM3_ESM.docx]

| Sup. Table 3. Unadjusted and Adjusted ordinal logistic regression of the association between background characteristics and ≥ 8 ANC visits in Nigeria, Mali, Guinea and Zambia DHS 2018 | | | | | | | | |
| --- | --- | --- | --- | --- | --- | --- | --- | --- |
| Variable | Nigeria (N=6709) | | Mali (N=1937) | | Guinea (N=1643) | | Zambia (N=1526) | |
|  | Crude OR  (95% CI) | AOR  (95% CI) | Crude OR  (95% CI) | AOR (95% CI) | Crude OR  (95% CI) | AOR  (95% CI) | Crude OR(95% CI) | AOR (95% CI) |
| **Labour force participation** |  |  |  |  |  |  |  |  |
| Low | Reference(1.0) | Reference(1.0) | Reference(1.0) | Reference(1.0) | Reference(1.0) | Reference(1.0) | Reference(1.0) | Reference(1.0) |
| Middle | 1.43(1.12-1.82)** | 1.04(0.79-1.38) | 0.30(0.10-0.94)** | 0.75(0.22-2.60) | 0.42(0.17-1.02)* | 0.72(0.26-1.96) | 2.77(0.95-8.03)* | 6.75(2.55-17.88)*** |
| High | 3.34(2.70-4.14)*** | 1.81(1.41-2.32)*** | 1.37(0.75-2.48) | 1.46(0.83-2.55) | 1.07(0.55-2.11) | 0.95(0.48-1.86) | 1.05(0.24-4.64) | 0.36(0.11-1.19) |
| **Disagreement with justification to wife beating** | |  |  |  |  |  |  |  |
| Low | Reference(1.0) | Reference(1.0) | Reference(1.0) | Reference(1.0) | Reference(1.0) | Reference(1.0) | Reference(1.0) | Reference(1.0) |
| Middle | 1.67(1.19-2.35)** | 0.93(0.65-1.34) | 3.00(1.41-6.41)** | 2.20(0.94-5.13)* | 1.06(0.45-2.52) | 0.99(0.42-2.33) | 2.76(0.63-12.11) | 3.60(0.49-26.49) |
| High | 4.34(3.23-5.84)*** | 1.34(0.98-1.83)* | 2.30(1.13-4.69)** | 1.82(0.85-3.87) | 0.94(0.41-2.18) | 0.64(0.28-1.47) | 2.09(0.57-7.70) | 1.91(0.24-15.28) |
| **Health decision making power** |  |  |  |  |  |  |  |  |
| Low | Reference(1.0) | Reference(1.0) | Reference(1.0) | Reference(1.0) | Reference(1.0) | Reference(1.0) | Reference(1.0) | Reference(1.0) |
| Middle | 0.90(0.69-1,16) | 0.70(0.51-0.94) | 3.40(1.02-11.37)** | 3.65(0.86-15.57)* | 1.14(0.39-3.31) | 0.58(0.23-1.48) | 1.50(0.22-10.24) | 1.56(0.24-10.19) |
| Highest | 1.61(1.26-2.06)*** | 0.95(0.69-1.31) | 6.64(2.47-17.81)*** | 4.59(1.27-16.57)** | 2.58(1.20-5.58)** | 0.65(0.24-1.75) | 0.95(0.28-3.26) | 1.53(0.31-7.67) |
| **Household decision making power** |  |  |  |  |  |  |  |  |
| Low | Reference(1.0) | Reference(1.0) | Reference(1.0) | Reference(1.0) | Reference(1.0) | Reference(1.0) | Reference(1.0) | Reference(1.0) |
| Middle | 2.12(1.72-2.63)*** | 1.38(1.09-1.73)** | 0.85(0.21-3.48) | 0.83(0.23-3.05) | 0.60(0.25-1.41) | 0.60(0.25-1.45) | 0.76(0.22-2.56) | 0.72(0.16-3.28) |
| High | 5.34(4.3-6.6)*** | 1.53(1.19-1.96)** | 1.28(0.73-2.25) | 0.68(0.56-0.82) | 0.76(0.36-1.58) | 0.80(0.34-1.89) | 0.62(0.18-2.12) | 0.39(0.08-1.93) |
| **Gender norm for sex negotiation** |  |  |  |  |  |  |  |  |
| Low | Reference(1.0) | Reference(1.0) | Reference(1.0) | Reference(1.0) | Reference(1.0) | Reference(1.0) | Reference(1.0) | Reference(1.0) |
| Middle | 2.38(1.84-3.08)*** | 1.07(0.80-1.44) | 2.04(0.92-4.57)* | 2.74(1.10-6.80)** | 1.53(0.73-3.18) | 1.64(0.69-3.93) | 3.12(0.47-20.78) | 1.86(0.35-9.80) |
| High | 4.51(3.64-5.60)*** | 1.35(1.07-1.70)** | 1.67(0.86-3.25) | 1.18(0.55-2.56) | 1.85(0.85-4.04) | 0.97(0.39-2.42) | 2.76(0.68-11.15) | 1.87(0.50-6.98) |
| **knowledge level of survival** |  |  |  |  |  |  |  |  |
| Low | Reference(1.0) | Reference(1.0) | Reference(1.0) | Reference(1.0) | Reference(1.0) | Reference(1.0) | Reference(1.0) | Reference(1.0) |
| Middle | 4.97(3.66-6.75)*** | 1.92(1.39-2.26)*** | 4.68(1.64-13.37)** | 3.84(1.19-12.39)** | 1.85(0.66-5.18) | 0.87(0.34-2.25) | 0.71(0.18-2.80) | 0.62(0.15-2.48) |
| High | 16.97(12.76-22.56)*** | 2.40(1.68-3.45)*** | 13.65(5.22-35.67)*** | 4.78(1.33-17.20)** | 5.77(2.64-12.63)*** | 1.75(0.55-5.62) | 1.46(0.45-4.77) | 0.39(0.06-2.61) |
| **Ownership of assets** |  |  |  |  |  |  |  |  |
| Low | Reference(1.0) | Reference(1.0) | Reference(1.0) | Reference(1.0) | Reference(1.0) | Reference(1.0) | Reference(1.0) | Reference(1.0) |
| Middle | 2.13(1.41-3.22)*** | 0.95(0.57-1.59) | 0.62(0.27-1.40) | 1.05(0.45-2.47) | 0.77(0.37-1.60) | 0.99(0.43-2.25) | 0.82(0.27-2.48) | 0.64(0.24-1.69) |
| High | 1.37(1.09-1.73)** | 0.84(0.64-1.11) | 0.82(0.43-1.55) | 2.13(1.01-4.51)** | 0.18(0.04-0.80)** | 0.38(0.06-2.36) | 1.58(0.32-7.68) | 1.71(0.40-7.34) |
| **Family planning** |  |  |  |  |  |  |  |  |
| Low | Reference(1.0) | Reference(1.0) | Reference(1.0) | Reference(1.0) | Reference(1.0) | Reference(1.0) | Reference(1.0) | Reference(1.0) |
| Middle | 1.34(1.03-1.75)** | 0.89(0.67-1.19) | 1.77(0.93-3.38)* | 1.25(0.63-2.50) | 1.16(0.48-2.80) | 0.70(0.27-1.81) | 1.13(0.39-3.31) | 1.02(0.33-3.20) |
| High | 3.03(2.51-3.65)*** | 1.26(1.02-1.56)** | 1.27(0.54-2.99) | 0.61(0.23-1.61) | 2.81(1.15-6.86)** | 1.27(0.47-3.43) | 1.34(0.36-5.03) | 0.67(0.11-4.16) |
| **Age at child birth (years)** |  |  |  |  |  |  |  |  |
| ≤19 | Reference(1.0) | Reference(1.0) | Reference(1.0) | Reference(1.0) | Reference(1.0) | Reference(1.0) | Reference(1.0) | Reference(1.0) |
| 20-24 | 2.12(1.46-3.07)*** | 1.47(0.98-2.21)* | 0.93(0.41-2.10) | 0.91(0.35-2.37) | 1.52(0.61-3.79) | 1.37(0.51-3.65) | 0.62(0.16-2.46) | 0.43(0.09-1.96) |
| 25-29 | 2.96(2.05-4.26)*** | 1.84(1.20-2.82)** | 0.67(0.31-1.46) | 0.79(0.29-2.18) | 1.20(0.45-3.21) | 1.38(0.46-4.15) | 0.29(0.07-1.18)* | 0.67(0.75-6.0) |
| 30-34 | 3.53(2.41-5.16)*** | 2.25(1.43-3.53)*** | 0.44(0.17-1.15)* | 0.76(0.20-2.82) | 1.29(0.46-3.58) | 1.81(0.49-6.72) | 0.59(0.12-2.80) | 2.80(0.48-16.15) |
| 35-39 | 2.87(1.94-4.26)*** | 2.18(1.35-3.52)** | 0.84(0.31-2.26) | 2.32(0.53-10.27) | 0.33(0.06-1.68) | 1.17(0.16-8.76) | 0.03(0.01-0.24)*** | 0.54(0.02-12.79) |
| ≥40 | 1.94(1.19-3.19)** | 2.26(1.17-4.39)** | 0.40(0.78-2.08) | 1.53(0.20-11.61) | 0.72(0.14-3.75) | 3.17(0.31-32.87) | 0.25(0.03-2.27) | 13.66(0.87-213.18)* |
| **Residence** |  |  |  |  |  |  |  |  |
| Urban | Reference(1.0) | Reference(1.0) | Reference(1.0) | Reference(1.0) | Reference(1.0) | Reference(1.0) | Reference(1.0) | Reference(1.0) |
| Rural | 0.24(0.20-0.29)*** | 0.65(0.51-0.81)*** | 0.25(0.14-0.45)*** | 1.05(0.46-2.40) | 0.15(0.08-0.30)*** | 0.76(0.28-2.07) | 0.79(0.27-2.29) | 0.70(0.13-3.77) |
| **Religion** |  |  |  |  |  |  |  |  |
| Christians | Reference(1.0) | Reference(1.0) | Reference(1.0) | Reference(1.0) | Reference(1.0) | Reference(1.0) | Reference(1.0) | Reference(1.0) |
| Muslim | 0.24(0.21-0.28)*** | 0.54(0.41-0.70)*** | 1.77(0.26-12.14) | 3.01(0.37-24.90) | 0.82(0.71-1.99) | 0.80(0.69-1.89) | 2.86(0.32-25.90) | 2.71(0.15-47.95) |
| Others | 0.13(0.04-0.38)*** | 0.39(0.08-1.91) | 0.81(0.05-12.58) | 5.79(30.0-111.95) | 0.80(0.64-2.10) | 0.75(0.62-1.91) | 0.44(0.05-3.63) | 0.41(0.03-5.30) |
| **Birth order** |  |  |  |  |  |  |  |  |
| 1-2 | Reference(1.0) | Reference(1.0) | Reference(1.0) | Reference(1.0) | Reference(1.0) | Reference(1.0) | Reference(1.0) | Reference(1.0) |
| 3-4 | 0.78(0.71-0.87)*** | 0.71(0.57-0.88)** | 0.68(0.35-1.31) | 0.69(0.31-1.55) | 1.05(0.53-2.06) | 1.13(0.51-2.52) | 0.13(0.03-0.58)** | 0.05(0.10-0.27)*** |
| ≥5 | 0.52(0.47-0.59)*** | 0.47(0.34-0.64)*** | 0.23(0.10-0.51)*** | 0.26(0.07-0.99)** | 0.18(0.06-0.55)** | 0.29(0.07-1.22)* | 0.05(0.001-0.28)** | 0.01(0.002-0.06)*** |
| **Pregnancy wanted** |  |  |  |  |  |  |  |  |
| No (later/no more) | Reference(1.0) | Reference(1.0) | Reference(1.0) | Reference(1.0) | Reference(1.0) | Reference(1.0) | Reference(1.0) | Reference(1.0) |
| Yes (then) | 0.67(0.54-0.84)*** | 1.02(0.77-1.35) | 1.49(0.65-3.39) | 1.04(0.42-2.63) | 4.44(1.00-19.72)** | 3.60(0.76-16.96) | 1.15(0.41-3.20) | 0.89(0.71-1.13) |
| **Polygyny** |  |  |  |  |  |  |  |  |
| Monogamous | Reference(1.0) | Reference(1.0) | Reference(1.0) | Reference(1.0) | Reference(1.0) | Reference(1.0) | Reference(1.0) | Reference(1.0) |
| Polygamous as first wife | 0.23(0.15-0.35)*** | 0.83(0.52-1.31) | 0.55(0.23-1.33) | 1.57(0.59-4.18) | 0.15(0.02-1.11)* | 0.37(0.04-3.69) | 1.94(0.45-8.26) | 4.34(0.83-22.78)* |
| Polygamous as 2^nd^ **or** higher | 0.36(0.28-0.46)*** | 1.01(0.76-1.34) | 0.90(0.44-1.84) | 1.63(0.67-3.95) | 1.02(0.49-2.14) | 1.34(0.55-3.28) | 1.90(0.50-8.30) | 4.30(0.80-23.10) |
| **Wealth quintiles** |  |  |  |  |  |  |  |  |
| Poorest | Reference(1.0) | Reference(1.0) | Reference(1.0) | Reference(1.0) | Reference(1.0) | Reference(1.0) | Reference(1.0) | Reference(1.0) |
| Poorer | 1.98(1.3-3.02)** | 1.31(0.83-2.06) | 1.24(0.27-5.55) | 1.00(0.22-4.63) | 0.32(0.04-2.88) | 0.27(0.03-2.38) | 1.83(0.34-9.94) | 1.25(0.25-6.28) |
| Middle | 4.55(2.99-6.92)*** | 1.66(1.06-2.61)** | 1.39(0.33-5.82) | 0.78(0.14-4.43) | 1.59(0.43-5.93) | 0.91(0.22-3.79) | 1.96(0.44-8.67) | 2.79(0.50-15.60) |
| Richer | 9.66(6.34-14.72)*** | 1.96(1.21-3.17)** | 3.29(0.90-12.09)* | 1.21(0.24-6.15) | 4.50(1.50-13.45)** | 1.53(0.41-5.65) | 1.17(0.20-6.99) | 1.61(0.15-17.96) |
| Richest | 20.99(13.86-31.79)*** | 2.43(1.46-4.04)** | 10.87(3.12-37.92)*** | 2.51(0.40-15.65) | 10.82(3.87-30.21)*** | 2.60(0.59-11.44) | 3.51(0.75-16.34) | 2.66(0.20-35.0) |
| **Distance to health facility** |  |  |  |  |  |  |  |  |
| big problem | Reference(1.0) | Reference(1.0) | Reference(1.0) | Reference(1.0) | Reference(1.0) | Reference(1.0) | Reference(1.0) | Reference(1.0) |
| Not a big problem | 1.36(1.09-1.69)** | 0.66(0.50-0.87)** | 3.44(1.51-7.82)** | 0.86(0.27-2.77) | 5.30(2.35-11.94)*** | 4.02(1.39-11.65)** | 0.60(0.24-1.49) | 0.28(0.06-1.37) |
| **Covered by Health Insurance** |  |  |  |  |  |  |  |  |
| No | Reference(1.0) | Reference(1.0) | Reference(1.0) | Reference(1.0) | Reference(1.0) | Reference(1.0) | Reference(1.0) | Reference(1.0) |
| Yes | 3.23(2.11-4.95)*** | 1.31(0.83-2.08) | 6.43(2.95-14.03)*** | 1.70(0.61-4.70) | 2.26(0.28-18.21) | 0.46(0.03-6.91) | 11.95(2.08-68.70)** | 11.4(1.92-67.55)** |
| **Husband level of education** |  |  |  |  |  |  |  |  |
| None | Reference(1.0) | Reference(1.0) | Reference(1.0) | Reference(1.0) | Reference(1.0) | Reference(1.0) | Reference(1.0) | Reference(1.0) |
| Primary | 4.43(3.13-6.26)*** | 1.71(1.15-2.54)** | 2.64(1.23-5.67)** | 1.55(0.70-3.42) | 0.35(0.05-2.68) | 0.24(0.03-1.98) | 3.04(0.31-29.94) | 1.74(0.17-17.79) |
| Secondary | 7.85(5.76-10.71)*** | 1.51(1.03-2.22)** | 3.97(1.98-7.95)*** | 1.31(0.52-3.34) | 1.86(0.77-4.50) | 1.02(0.38-2.69) | 9.71(1.23-76.73)** | 8.36(0.99-70.39)* |
| Higher | 10.86(7.77-15.18)*** | 1.51(1.00-2.30)* | 12.61(5.22-30.51)*** | 2.93(0.95-9.04)* | 4.93(2.12-11.44)*** | 1.15(0.45-2.92) | 32.67(3.54-301.45)** | 42.78(1.65-110.8)** |
| **Difference in age between husband and wife** |  |  |  |  |  |  |  |  |
| Wife older or same age | Reference(1.0) | Reference(1.0) | Reference(1.0) | Reference(1.0) | Reference(1.0) | Reference(1.0) | Reference(1.0) | Reference(1.0) |
| Husband 1-5 years older | 1.00(0.59-1.68) | 1.14(0.65-1.99) | 0.40(0.11-1.42) | 0.34(0.07-1.56) | 1.00(1.00-1.00) | 1.00(1.00-1.00) | 1.30(0.26-6.42) | 1.19(0.07-20.24) |
| Husband 6-10 years older | 0.59(0.35-1.01)* | 0.99(0.57-1.74) | 0.31(0.09-1.13)* | 0.26(0.06-1.08)* | 0.81(0.30-2.21) | 0.61(0.20-1.90) | 0.69(0.12-4.01) | 0.66(0.04-11.63) |
| Husband > 10 years older | 0.40(0.23-0.69)*** | 0.95(0.53-1.71) | 0.34(0.10-1.14)* | 0.23(0.06-0.93)** | 1.15(0.49-2.70) | 1.00(0.35-2.88) | 0.51(0.04-5.92) | 0.51(0.01-19.56) |

***p<0.001,**p<0.05,*p<0.10
