# Supplementary material for: Temporal relationship between Women’s empowerment and utilization of antenatal care services: lessons from four National Surveys in sub-Saharan Africa
Source: BMC Pregnancy Childbirth. 2021 Mar 10;21:198. doi: 10.1186/s12884-021-03679-8 (PMC7944901; doi:10.1186/s12884-021-03679-8)
Supplement: Supplementary file 4 — Additional file 4. Unadjusted and Adjusted ordinal logistic regression of the association between background characteristics and ≥ 4 ANC visits in Nigeria, Mali, Guinea and Zambia DHS 2018. [file 12884_2021_3679_MOESM4_ESM.docx]

| Sup. Table 4. Unadjusted and Adjusted ordinal logistic regression of the association between background characteristics and ≥ 4 ANC visits in Nigeria, Mali, Guinea and Zambia DHS 2018 | | | | | | | | |
| --- | --- | --- | --- | --- | --- | --- | --- | --- |
| Variable | Nigeria (N=6709) | | Mali (N=1937) | | Guinea (N=1643) | | Zambia (N=1526) | |
|  | Crude OR  (95% CI) | AOR  (95% CI) | Crude OR  (95% CI) | AOR (95% CI) | Crude OR  (95% CI) | AOR  (95% CI) | Crude OR(95% CI) | AOR (95% CI) |
| **Labour force participation** |  |  |  |  |  |  |  |  |
| Low | Reference(1.0) | Reference(1.0) | Reference(1.0) | Reference(1.0) | Reference(1.0) | Reference(1.0) | Reference(1.0) | Reference(1.0) |
| Middle | 1.42(1.22-1.65)*** | 1.21(1.02-1.43)** | 0.63(0.48-0.84)** | 1.10(0.81-1.48) | 0.82(0.60-1.12) | 0.90(0.65-1.25) | 1.35(0.98-1.87)* | 1.21(2.55-17.88) |
| High | 2.58(2.18-3.04)*** | 1.55(1.28-1.88)*** | 1.32(1.03-1.70)** | 1.06(0.79-1.43) | 2.05(1.55-2.70)*** | 1.65(1.23-2.23)** | 0.98(0.72-1.33) | 0.95(0.69-1.31) |
| **Disagreement with justification to wife beating** | |  |  |  |  |  |  |  |
| Low | Reference(1.0) | Reference(1.0) | Reference(1.0) | Reference(1.0) | Reference(1.0) | Reference(1.0) | Reference(1.0) | Reference(1.0) |
| Middle | 1.44(1.15-1.80)** | 1.03(0.82-1.30) | 1.29(1.00-1.66)** | 0.91(0.68-1.22) | 0.95(0.70-1.29) | 0.80(0.57-1.12) | 1.08(0.74-1.57) | 1.02(0.70-1.49) |
| High | 2.65(2.16-3.24)*** | 1.27(1.03-1.56)** | 1.31(1.03-1.68)** | 1.04(0.78-1.38) | 0.81(0.59-1.11) | 0.67(0.49-0.92)** | 1.20(0.86-1.67) | 1.18(0.83-1.68) |
| **Health decision making power** |  |  |  |  |  |  |  |  |
| Low | Reference(1.0) | Reference(1.0) | Reference(1.0) | Reference(1.0) | Reference(1.0) | Reference(1.0) | Reference(1.0) | Reference(1.0) |
| Middle | 1.63(1.36-1.95)*** | 1.18(0.97-1.43) | 1.44(1.06-1.95)** | 1.22(0.87-1.71) | 1.38(1.01-1.90)** | 1.09(0.79-1.51) | 1.12(0.73-1.73) | 1.16(0.72-1.84) |
| Highest | 2.55(2.12-3.07)*** | 1.31(1.05-1.64)** | 2.32(1.76-3.06)*** | 1.29(0.90-1.86) | 2.08(1.53-2.81)*** | 1.39(0.96-2.00)* | 1.08(0.78-1.51) | 1.09(0.74-1.61) |
| **Household decision making power** |  |  |  |  |  |  |  |  |
| Low | Reference(1.0) | Reference(1.0) | Reference(1.0) | Reference(1.0) | Reference(1.0) | Reference(1.0) | Reference(1.0) | Reference(1.0) |
| Middle | 1.51(1.30-1.76)*** | 1.12(0.94-1.34) | 1.69(0.98-2.94)* | 1.38(0.76-2.52) | 1.49(1.11-2.00)** | 1.41(1.03-1.92)** | 0.86(0.66-1.12) | 0.87(0.66-1.16) |
| High | 3.38(2.84-4.01)*** | 1.31(1.07-1.60)** | 1.61(1.27-2.03)*** | 1.13(0.86-1.50) | 1.96(1.48-2.58)*** | 1.57(1.12-2.19)** | 0.78(0.56-1.09) | 0.81(0.57-1.15) |
| **Gender norm for sex negotiation** |  |  |  |  |  |  |  |  |
| Low | Reference(1.0) | Reference(1.0) | Reference(1.0) | Reference(1.0) | Reference(1.0) | Reference(1.0) | Reference(1.0) | Reference(1.0) |
| Middle | 1.90(1.59-2.27)*** | 1.12(0.93-1.35) | 1.27(0.92-1.75) | 1.30(0.90-1.87) | 1.57(1.15-2.14)** | 1.40(1.01-1.94)** | 2.23(1.54-3.21)*** | 2.18(1.50-3.16)*** |
| High | 2.95(2.53-3.45)*** | 1.14(0.96-1.34) | 1.35(1.03-1.77)* | 1.08(0.82-1.42) | 2.52(1.86-3.40)*** | 1.57(1.12-2.19)** | 1.76(1.30-2.39)*** | 1.66(1.23-2.24)** |
| **knowledge level of survival** |  |  |  |  |  |  |  |  |
| Low | Reference(1.0) | Reference(1.0) | Reference(1.0) | Reference(1.0) | Reference(1.0) | Reference(1.0) | Reference(1.0) | Reference(1.0) |
| Middle | 2.78(2.35-3.28)*** | 1.44(1.19-1.72)*** | 1.87(1.44-2.43)*** | 1.40(1.06-1.85)** | 1.53(1.14-2.05)** | 0.99(0.74-1.33) | 1.14(0.80-1.61) | 1.18(0.81-1.72) |
| High | 10.90(9.09-13.08)*** | 2.52(1.98-3.20)*** | 5.24(3.92-7.00)*** | 2.37(1.66-3.39)*** | 2.95(2.20-3.95)*** | 1.18(0.83-1.69) | 1.12(0.82-1.52) | 0.99(0.66-1.48) |
| **Ownership of assets** |  |  |  |  |  |  |  |  |
| Low | Reference(1.0) | Reference(1.0) | Reference(1.0) | Reference(1.0) | Reference(1.0) | Reference(1.0) | Reference(1.0) | Reference(1.0) |
| Middle | 2.23(1.44-3.45)*** | 1.18(0.77-1.83) | 0.71(0.53-0.95)** | 1.04(0.75-1.45) | 0.86(0.63-1.17) | 1.12(0.84-1.51) | 1.47(1.07-2.01)** | 1.37(0.98-1.93)* |
| High | 1.41(1.17-1.71)*** | 1.02(0.83-1.25) | 0.85(0.65-1.11) | 1.49(1.14-1.96)** | 0.65(0.48-0.90)** | 1.05(0.73-1.49) | 1.66(1.09-2.51)** | 1.61(1.06-2.45)** |
| **Family planning** |  |  |  |  |  |  |  |  |
| Low | Reference(1.0) | Reference(1.0) | Reference(1.0) | Reference(1.0) | Reference(1.0) | Reference(1.0) | Reference(1.0) | Reference(1.0) |
| Middle | 1.29(1.08-1.53)** | 1.04(0.85-1.27) | 1.32(1.05-1.67)** | 1.12(0.86-1.44) | 1.68(1.25-2.27)** | 1.43(1.06-1.94)** | 0.94(0.69-1.29) | 0.90(0.65-1.24) |
| High | 3.13(2.71-3.61)*** | 1.52(1.30-1.78)*** | 1.26(0.89-1.78) | 0.82(0.56-1.21) | 2.60(1.86-3.64)*** | 1.76(1.22-2.53)*** | 1.47(0.97-2.23)* | 1.23(0.78-1.93) |
| **Age at child birth (years)** |  |  |  |  |  |  |  |  |
| ≤19 | Reference(1.0) | Reference(1.0) | Reference(1.0) | Reference(1.0) | Reference(1.0) | Reference(1.0) | Reference(1.0) | Reference(1.0) |
| 20-24 | 1.61(1.30-1.98)*** | 1.28(1.00-1.64)* | 1.06(0.76-1.47) | 1.26(0.86-1.84) | 1.48(1.01-2.16)** | 1.39(0.92-2.10) | 1.00(0.67-1.51) | 1.01(0.63-1.62) |
| 25-29 | 1.63(1.31-2.02)*** | 1.20(0.91-1.59) | 0.88(0.63-1.24) | 1.04(0.63-1.73) | 1.07(0.74-1.55) | 1.14(0.72-1.82) | 0.77(0.51-1.15) | 0.98(0.54-1.80) |
| 30-34 | 1.89(1.49-2.40)*** | 1.52(1.10-2.09)** | 0.83(0.58-1.18) | 1.17(0.67-2.04) | 0.99(0.66-1.48) | 1.27(0.76-2.12) | 0.86(0.55-1.33) | 1.28(0.64-2.5) |
| 35-39 | 1.38(1.06-1.79)** | 1.03(0.74-1.43) | 1.21(0.83-1.76) | 1.88(1.03-3.42) | 0.75(0.48-1.17) | 1.00(0.56-1.79) | 0.76(0.45-1.28) | 1.43(0.68-3.01) |
| ≥40 | 1.14(0.85-1.54) | 1.26(0.86-1.84) | 0.79(0.47-1.33) | 1.32(0.64-2.74) | 1.13(0.63-2.03) | 1.52(0.75-3.07) | 1.04(0.58-1.88) | 1.87(0.79-4.45) |
| **Residence** |  |  |  |  |  |  |  |  |
| Urban | Reference(1.0) | Reference(1.0) | Reference(1.0) | Reference(1.0) | Reference(1.0) | Reference(1.0) | Reference(1.0) | Reference(1.0) |
| Rural | 0.31(0.26-0.36)*** | 0.87(0.71-1.05) | 0.23(0.16-0.32)*** | 0.80(0.48-1.35) | 0.35(0.26-0.48)*** | 0.96(0.62-1.47) | 1.26(0.92-1.73) | 1.34(0.80-2.24) |
| **Religion** |  |  |  |  |  |  |  |  |
| Christians | Reference(1.0) | Reference(1.0) | Reference(1.0) | Reference(1.0) | Reference(1.0) | Reference(1.0) | Reference(1.0) | Reference(1.0) |
| Muslim | 0.20(0.16-0.24)*** | 0.75(0.61-0.92)** | 0.87(0.47-1.62) | 0.79(0.44-1.41) | 1.55(0.98-2.46)* | 1.47(0.91-2.36) | 0.67(0.14-3.15) | 0.58(0.12-2.87) |
| Others | 0.18(0.03-0.91)** | 0.48(0.18-1.26) | 0.56(0.23-1.37) | 0.99(0.45-2.20) | 1.87(0.87-4.00) | 1.71(0.70-4.20) | 1.10(0.36-3.40) | 1.50(0.52-4.36) |
| **Birth order** |  |  |  |  |  |  |  |  |
| 1-2 | Reference(1.0) | Reference(1.0) | Reference(1.0) | Reference(1.0) | Reference(1.0) | Reference(1.0) | Reference(1.0) | Reference(1.0) |
| 3-4 | 0.73(0.64-0.84)*** | 0.72(0.60-0.87)** | 0.76(0.60-0.96)** | 0.85(0.59-1.24) | 0.87(0.67-1.13) | 0.84(0.58-1.21) | 0.81(0.60-1.08) | 0.71(0.49-1.05)* |
| ≥5 | 0.46(0.40-0.52)*** | 0.62(0.50-0.78)*** | 0.64(0.51-0.80)*** | 0.91(0.59-1.41) | 0.65(0.49-0.87)** | 0.69(0.43-1.11) | 0.67(0.51-0.89)** | 0.49(0.29-0.84)** |
| **Pregnancy wanted** |  |  |  |  |  |  |  |  |
| No (later/no more) | Reference(1.0) | Reference(1.0) | Reference(1.0) | Reference(1.0) | Reference(1.0) | Reference(1.0) | Reference(1.0) | Reference(1.0) |
| Yes (then) | 0.84(0.69-1.01)* | 1.58(1.24-2.00)*** | 1.43(1.07-1.91)** | 1.49(1.07-2.07)** | 1.27(0.91-1.78) | 0.93(0.64-1.34) | 1.28(1.00-1.64)* | 1.25(0.95-1.64) |
| **Polygyny** |  |  |  |  |  |  |  |  |
| Monogamous | Reference(1.0) | Reference(1.0) | Reference(1.0) | Reference(1.0) | Reference(1.0) | Reference(1.0) | Reference(1.0) | Reference(1.0) |
| Polygamous as first wife | 0.46(0.37-0.57)*** | 1.09(0.86-1.39) | 0.58(0.43-0.79)*** | 0.77(0.53-1.11) | 0.80(0.54-1.17) | 1.04(0.69-1.56) | 0.85(0.49-1.47) | 1.10(0.57-2.12) |
| Polygamous as 2^nd^ **or** higher | 0.51(0.43-0.59)*** | 0.93(0.77-1.12) | 1.00(0.78-1.29) | 1.12(0.83-1.52) | 0.60(0.45-0.78)*** | 0.65(0.48-0.90)** | 1.34(0.74-2.45) | 1.44(0.82-2.56) |
| **Wealth quintiles** |  |  |  |  |  |  |  |  |
| Poorest | Reference(1.0) | Reference(1.0) | Reference(1.0) | Reference(1.0) | Reference(1.0) | Reference(1.0) | Reference(1.0) | Reference(1.0) |
| Poorer | 1.73(1.42-2.09)*** | 1.26(1.02-1.54)** | 1.66(1.14-2.42)** | 1.62(1.10-2.40)** | 1.59(1.04-2.43)** | 1.39(0.91-2.12) | 0.92(0.64-1.32) | 0.87(0.60-1.25) |
| Middle | 3.25(2.64-4.00)*** | 1.48(1.19-1.85)*** | 1.88(1.30-2.73)** | 1.61(1.08-2.38)** | 2.54(1.67-3.86)*** | 2.09(1.33-3.28)** | 0.85(0.59-1.23) | 0.85(0.58-1.23) |
| Richer | 6.01(4.74-7.60)*** | 1.61(1.22-2.11)** | 3.76(2.54-5.58)*** | 2.38(1.52-3.73)*** | 4.51(3.01-6.76)*** | 2.49(1.50-4.12)*** | 0.52(0.35-0.76)** | 0.56(0.33-0.93)** |
| Richest | 15.98(12.20-20.93)*** | 2.43(1.73-3.40)*** | 12.1(8.18-18.04)*** | 4.76(2.48-9.11)*** | 6.46(4.02-10.39)*** | 3.18(1.59-6.34)** | 1.23(0.80-1.90) | 1.06(0.52-2.14) |
| **Distance to health facility** |  |  |  |  |  |  |  |  |
| big problem | Reference(1.0) | Reference(1.0) | Reference(1.0) | Reference(1.0) | Reference(1.0) | Reference(1.0) | Reference(1.0) | Reference(1.0) |
| Not a big problem | 2.09(1.79-2.44)** | 1.13(0.95-1.35) | 2.36(1.86-3.00)*** | 1.45(1.03-2.02)** | 1.92(1.50-2.46)*** | 1.03(0.75-1.42) | 1.04(0.79-1.37) | 1.05(0.75-1.48) |
| **Covered by Health Insurance** |  |  |  |  |  |  |  |  |
| No | Reference(1.0) | Reference(1.0) | Reference(1.0) | Reference(1.0) | Reference(1.0) | Reference(1.0) | Reference(1.0) | Reference(1.0) |
| Yes | 2.74(1.50-5.03)*** | 0.92(0.57-1.48) | 5.28(3.24-8.59)*** | 1.26(0.53-3.03) | 2.28(0.87-5.96)* | 0.57(0.15-2.23) | 1.33(0.46-3.83) | 0.48(0.15-1.56) |
| **Husband level of education** |  |  |  |  |  |  |  |  |
| None | Reference(1.0) | Reference(1.0) | Reference(1.0) | Reference(1.0) | Reference(1.0) | Reference(1.0) | Reference(1.0) | Reference(1.0) |
| Primary | 2.70(2.19-3.31)*** | 1.48(1.20-1.83)*** | 1.57(1.13-2.18)** | 0.99(0.67-1.44) | 1.75(1.06-2.90)** | 1.42(0.81-2.48) | 0.85(0.54-1.34) | 0.84(0.52-1.36) |
| Secondary | 4.97(4.21-5.88)*** | 1.59(1.30-1.93)*** | 3.18(2.31-4.36)*** | 1.37(0.92-2.05) | 2.36(1.64-3.38)*** | 1.55(1.02-2.35)** | 0.96(0.60-1.55) | 1.09(0.64-1.83) |
| Higher | 7.98(6.43-9.92)*** | 1.70(1.30-2.21)*** | 9.71(5.11-18.42)*** | 2.49(1.27-4.87)** | 3.19(2.03-5.00)*** | 1.25(0.71-2.19) | 2.12(1.09-4.15)** | 2.38(1.03-5.48)** |
| **Difference in age between husband and wife** |  |  |  |  |  |  |  |  |
| Wife older or same age | Reference(1.0) | Reference(1.0) | Reference(1.0) | Reference(1.0) | Reference(1.0) | Reference(1.0) | Reference(1.0) | Reference(1.0) |
| Husband 1-5 years older | 1.19(0.76-1.85) | 1.40(0.83-2.38) | 0.30(0.14-0.68)** | 0.25(0.10-0.61)** | 5.69(1.13-28.74)** | 6.82(0.70-66.84)* | 0.86(0.44-1.70) | 0.90(0.44-1.82) |
| Husband 6-10 years older | 0.90(0.59-1.39) | 1.42(0.85-2.37) | 0.34(0.15-0.74)** | 0.29(0.12-0.69)** | 5.22(1.11-24.68)** | 6.45(0.73-56.63)* | 0.74(0.36-1.51) | 0.79(0.38-1.65) |
| Husband > 10 years older | 0.74(0.48-1.14) | 1.47(0.87-2.48) | 0.41(0.19-0.90)** | 0.32(0.14-0.74)** | 5.18(1.08-24.81)** | 8.03(0.89-72.45)* | 1.08(0.51-2.28) | 1.19(0.54-2.61) |

***p<0.001,**p<0.05,*p<0.10
