# Supplementary material for: Temporal relationship between Women’s empowerment and utilization of antenatal care services: lessons from four National Surveys in sub-Saharan Africa
Source: BMC Pregnancy Childbirth. 2021 Mar 10;21:198. doi: 10.1186/s12884-021-03679-8 (PMC7944901; doi:10.1186/s12884-021-03679-8)
Supplement: Supplementary file 5 — Additional file 5. Unadjusted and Adjusted ordinal logistic regression of the association between background characteristics and early timing of ANC visits (≤ 3 months gestation) in Nigeria, Mali, Guinea and Zambia DHS 2018. [file 12884_2021_3679_MOESM5_ESM.docx]

| Sup. Table 5. Unadjusted and Adjusted ordinal logistic regression of the association between background characteristics and early timing of ANC visits (≤3 months gestation) in Nigeria, Mali, Guinea and Zambia DHS 2018 | | | | | | | | |
| --- | --- | --- | --- | --- | --- | --- | --- | --- |
| Variable | Nigeria (N=6709) | | Mali (N=1937) | | Guinea (N=1637) | | Zambia (N=1526) | |
|  | Crude OR  (95% CI) | AOR  (95% CI) | Crude OR  (95% CI) | AOR (95% CI) | Crude OR  (95% CI) | AOR  (95% CI) | Crude OR(95% CI) | AOR (95% CI) |
| **Labour force participation** |  |  |  |  |  |  |  |  |
| Low | Reference(1.0) | Reference(1.0) | Reference(1.0) | Reference(1.0) | Reference(1.0) | Reference(1.0) | Reference(1.0) | Reference(1.0) |
| Middle | 1.47(1.20-1.79)*** | 1.23(1.00-1.53)* | 0.59(0.45-0.78)*** | 0.96(0.70-1.32) | 0.82(0.58-1.14) | 0.92(0.64-1.33) | 1.28(0.92-1.77) | 1.07(0.76-1.49) |
| High | 2.07(1.69-2.54)*** | 1.47(1.18-1.82)** | 1.25(0.98-1.59)* | 1.17(0.89-1.52) | 1.31(0.98-1.76)* | 1.16(0.83-1.62) | 0.78(0.57-1.07) | 0.85(0.61-1.17) |
| **Disagreement with justification to wife beating** | |  |  |  |  |  |  |  |
| Low | Reference(1.0) | Reference(1.0) | Reference(1.0) | Reference(1.0) | Reference(1.0) | Reference(1.0) | Reference(1.0) | Reference(1.0) |
| Middle | 1.41(1.05-1.89)** | 1.06(0.78-1.43) | 1.26(0.98-1.64)* | 0.98(0.72-1.32) | 0.69(0.50-0.95)** | 0.71(0.50-0.99)** | 0.96(0.67-1.36) | 0.93(0.64-1.34) |
| High | 2.24(1.78-2.82)*** | 1.21(0.95-1.53) | 1.34(1.04-1.73)** | 1.15(0.87-1.52) | 0.98(0.71-1.35) | 1.00(0.72-1.39) | 0.90(0.66-1.23) | 0.99(0.71-1.40) |
| **Health decision making power** |  |  |  |  |  |  |  |  |
| Low | Reference(1.0) | Reference(1.0) | Reference(1.0) | Reference(1.0) | Reference(1.0) | Reference(1.0) | Reference(1.0) | Reference(1.0) |
| Middle | 1.09(0.86-1.39) | 0.93(0.72-1.20) | 1.17(0.83-1.65) | 1.06(0.73-1.54) | 1.40(1.02-1.93)** | 1.29(0.92-1.80) | 0.80(0.50-1.29) | 0.92(0.57-1.49) |
| High | 1.41(1.13-1.77)** | 1.02(0.78-1.33) | 2.01(1.53-2.65)*** | 1.43(1.00-2.03) | 1.54(1.12-2.10)*** | 1.14(0.78-1.66) | 0.78(0.56-1.09) | 1.02(0.68-1.52) |
| **Household decision making power** |  |  |  |  |  |  |  |  |
| Low | Reference(1.0) | Reference(1.0) | Reference(1.0) | Reference(1.0) | Reference(1.0) | Reference(1.0) | Reference(1.0) | Reference(1.0) |
| Middle | 1.41(1.16-1.72)** | 1.05(0.85-1.31) | 1.36(0.82-2.23) | 1.22(0.68-2.21) | 0.94(0.70-1.28) | 0.92(0.67-1.26) | 0.65(0.48-0.88)** | 0.72(0.52-0.99)** |
| High | 2.52(2.08-3.03)*** | 1.09(0.88-1.35) | 1.37(1.09-1.72)** | 1.00(0.78-1.28) | 1.06(0.79-1.42) | 0.99(0.73-1.34) | 0.53(0.37-0.77)** | 0.63(0.43-0.92)** |
| **Gender norm for sex negotiation** |  |  |  |  |  |  |  |  |
| Low | Reference(1.0) | Reference(1.0) | Reference(1.0) | Reference(1.0) | Reference(1.0) | Reference(1.0) | Reference(1.0) | Reference(1.0) |
| Middle | 2.18(1.75-2.73)*** | 1.47(1.15-1.86)** | 1.45(1.02-2.05)** | 1.54(1.04-2.30)** | 0.98(0.70-1.36) | 0.91(0.65-1.26) | 1.05(0.71-1.56) | 1.04(0.71-1.53) |
| High | 2.59(2.16-3.11)*** | 1.32(1.09-1.61)** | 1.32(1.04-1.68)** | 1.06(0.81-1.38) | 1.53(1.14-2.06)** | 1.22(0.88-1.70) | 1.20(0.87-1.64) | 1.18(0.87-1.61) |
| **Knowledge level of survival** |  |  |  |  |  |  |  |  |
| Low | Reference(1.0) | Reference(1.0) | Reference(1.0) | Reference(1.0) | Reference(1.0) | Reference(1.0) | Reference(1.0) | Reference(1.0) |
| Middle | 2.14(1.70-2.70)*** | 1.19(0.91-1.55) | 1.43(1.11-1.86)** | 1.14(0.85-1.52) | 1.51(1.12-2.04)** | 1.13(0.82-1.55) | 0.92(0.67-1.25) | 1.08(0.77-1.50) |
| High | 4.38(3.55-5.42)*** | 1.40(1.01-1.92)** | 3.87(2.98-5.02)*** | 1.75(1.27-2.41)** | 1.86(1.35-2.56)*** | 1.00(0.66-1.53) | 0.80(0.58-1.10) | 0.93(0.62-1.39) |
| **Ownership of assets** |  |  |  |  |  |  |  |  |
| Low | Reference(1.0) | Reference(1.0) | Reference(1.0) | Reference(1.0) | Reference(1.0) | Reference(1.0) | Reference(1.0) | Reference(1.0) |
| Middle | 2.53(1.69-3.79)*** | 1.68(1.14-2.48)** | 0.74(0.53-1.03)* | 1.00(0.69-1.45) | 0.83(0.62-1.10) | 1.02(0.76-1.38) | 1.59(1.21-2.08)** | 1.47(1.10-1.96)** |
| High | 1.36(1.10-1.67)** | 1.04(0.83-1.30) | 0.85(0.67-1.09) | 1.31(1.01-1.70)** | 0.53(0.38-0.74)*** | 0.72(0.50-1.02)* | 1.63(1.09-2.43)** | 1.63(1.06-2.52)** |
| **Family planning** |  |  |  |  |  |  |  |  |
| Low | Reference(1.0) | Reference(1.0) | Reference(1.0) | Reference(1.0) | Reference(1.0) | Reference(1.0) | Reference(1.0) | Reference(1.0) |
| Middle | 1.29(1.03-1.61)** | 1.20(0.94-1.54) | 1.06(0.85-1.32) | 0.93(0.72-1.21) | 1.59(1.17-2.15)** | 1.54(1.10-2.16)** | 0.86(0.64-1.15) | 0.79(0.58-1.09) |
| High | 1.97(1.67-2.32)*** | 1.24(1.04-1.47)** | 1.31(0.92-1.87) | 1.10(0.76-1.58) | 1.99(1.41-2.81)*** | 1.52(1.04-2.20)** | 1.17(0.79-1.74) | 1.21(0.79-1.85) |
| **Age at child birth (years)** |  |  |  |  |  |  |  |  |
| ≤19 | Reference(1.0) | Reference(1.0) | Reference(1.0) | Reference(1.0) | Reference(1.0) | Reference(1.0) | Reference(1.0) | Reference(1.0) |
| 20-24 | 1.33(1.02-1.74)** | 1.10(0.83-1.47) | 1.01(0.73-1.41) | 1.26(0.88-1.83) | 1.45(0.94-2.23)* | 1.31(0.83-2.08) | 1.03(0.67-1.56) | 1.06(0.67-1.66) |
| 25-29 | 1.42(1.08-1.87)** | 1.14(0.82-1.59) | 0.79(0.56-1.13) | 1.19(0.77-1.86) | 1.20(0.82-1.78) | 1.19(0.75-1.88) | 0.71(0.45-1.10) | 0.84(0.47-1.49) |
| 30-34 | 1.46(1.09-1.95)** | 1.24(0.83-1.86) | 0.64(0.44-0.94)** | 2.00(0.69-2.09) | 1.51(1.01-2.26)** | 1.55(0.94-2.56) | 0.68(0.44-1.06)* | 0.94(0.49-1.78) |
| 35-39 | 1.14(0.83-1.56) | 1.09(0.72-1.65) | 0.85(0.56-1.30) | 1.89(1.04-3.43)** | 1.08(0.68-1.71) | 1.13(0.62-2.05) | 0.51(0.27-0.95)** | 0.82(0.40-1.66) |
| ≥40 | 0.74(0.47-1.18) | 0.94(0.54-1.65) | 0.52(0.29-0.93)** | 1.21(0.60-2.47) | 0.95(0.48-1.86) | 0.88(0.35-2.20) | 0.62(0.35-1.08)* | 0.80(0.36-1.78) |
| **Residence** |  |  |  |  |  |  |  |  |
| Urban | Reference(1.0) | Reference(1.0) | Reference(1.0) | Reference(1.0) | Reference(1.0) | Reference(1.0) | Reference(1.0) | Reference(1.0) |
| Rural | 0.55(0.46-0.66)*** | 1.12(0.92-1.38) | 0.44(0.34-0.56)*** | 1.64(1.10-2.44)** | 0.55(0.40-0.77)*** | 1.44(0.90-2.32) | 1.75(1.28-2.40)** | 1.84(1.20-2.84)** |
| **Religion** |  |  |  |  |  |  |  |  |
| Christians | Reference(1.0) | Reference(1.0) | Reference(1.0) | Reference(1.0) | Reference(1.0) | Reference(1.0) | Reference(1.0) | Reference(1.0) |
| Muslim | 0.35(0.29-0.42)*** | 0.69(0.53-0.88)** | 1.09(0.63-1.88) | 1.34(0.71-2.52) | 4.51(2.18-9.34)*** | 4.46(2.16-9.23)*** | 0.89(0.18-4.33) | 0.78(0.17-3.61) |
| Others | 0.25(0.05-1.21)* | 0.37(0.10-1.42) | 0.66(0.31-1.40) | 1.49(0.67-3.31) | 0.86(0.13-5.50) | 0.85(0.12-6.05) | 1.32(0.34-5.02) | 1.63(0.36-7.34) |
| **Birth order** |  |  |  |  |  |  |  |  |
| 1-2 | Reference(1.0) | Reference(1.0) | Reference(1.0) | Reference(1.0) | Reference(1.0) | Reference(1.0) | Reference(1.0) | Reference(1.0) |
| 3-4 | 0.74(0.62-0.88)** | 0.77(0.62-0.95)** | 0.68(0.53-0.88)** | 0.71(0.51-1.00)** | 0.96(0.74-1.25) | 0.94(0.68-1.30) | 0.73(0.56-0.96)** | 0.73(0.52-1.02)* |
| ≥5 | 0.42(0.34-0.51)*** | 0.58(0.42-0.80)** | 0.45(0.34-0.59)*** | 0.59(0.38-0.92)** | 0.97(0.74-1.27) | 1.08(0.70-1.66) | 0.60(0.44-0.81)** | 0.56(0.34-0.93)** |
| **Pregnancy wanted** |  |  |  |  |  |  |  |  |
| No (later/no more) | Reference(1.0) | Reference(1.0) | Reference(1.0) | Reference(1.0) | Reference(1.0) | Reference(1.0) | Reference(1.0) | Reference(1.0) |
| Yes (then) | 1.13(0.88-1.45) | 1.70(1.29-2.24)*** | 1.82(1.35-2.44)*** | 1.78(1.31-2.44)*** | 1.19(0.85-1.66) | 0.98(0.68-1.42) | 1.08(0.83-1.41) | 1.07(0.80-1.44) |
| **Polygyny** |  |  |  |  |  |  |  |  |
| Monogamous | Reference(1.0) | Reference(1.0) | Reference(1.0) | Reference(1.0) | Reference(1.0) | Reference(1.0) | Reference(1.0) | Reference(1.0) |
| Polygamous as first wife | 0.42(0.31-0.56)*** | 0.97(0.71-1.33) | 0.51(0.37-0.72)*** | 0.73(0.49-1.10) | 0.89(0.60-1.33) | 0.95(0.62-1.47) | 1.26(0.63-2.50) | 1.48(0.78-2.82) |
| Polygamous as 2^nd^ **or** higher | 0.55(0.43-0.68)*** | 0.93(0.72-1.21) | 1.03(0.81-1.31) | 1.22(0.92-1.61) | 0.82(0.59-1.14) | 0.78(0.55-1.09) | 1.38(0.70-2.70) | 1.25(0.65-2.44) |
| **Wealth quintiles** |  |  |  |  |  |  |  |  |
| Poorest | Reference(1.0) | Reference(1.0) | Reference(1.0) | Reference(1.0) | Reference(1.0) | Reference(1.0) | Reference(1.0) | Reference(1.0) |
| Poorer | 2.01(1.53-2.64)*** | 1.61(1.21-2.15)** | 1.16(0.80-1.69) | 1.11(0.74-1.66) | 1.62(1.11-2.37)** | 1.53(1.05-2.25)** | 0.70(0.51-0.97)** | 0.72(0.51-1.03)* |
| Middle | 2.86(2.16-3.78)*** | 1.75(1.28-2.41)** | 1.65(1.16-2.33)** | 1.44(0.97-2.14)* | 1.95(1.27-3.00)** | 1.71(1.11-2.65)** | 0.58(0.41-0.82)** | 0.83(0.57-1.19) |
| Richer | 3.37(2.55-4.46)*** | 1.59(1.12-2.25)** | 2.50(1.76-3.55)*** | 1.85(1.17-2.93)** | 2.74(1.80-4.17)*** | 2.05(1.19-3.50)** | 0.47(0.31-0.71)*** | 1.01(0.56-1.83) |
| Richest | 6.70(5.02-8.94)*** | 2.52(1.67-3.82)*** | 5.46(3.74-7.97)*** | 3.45(1.79-6.63)*** | 4.13(2.52-6.78)*** | 3.04(1.54-5.97)** | 0.69(0.46-1.02)* | 1.63(0.82-3.26) |
| **Distance to health facility** |  |  |  |  |  |  |  |  |
| big problem | Reference(1.0) | Reference(1.0) | Reference(1.0) | Reference(1.0) | Reference(1.0) | Reference(1.0) | Reference(1.0) | Reference(1.0) |
| Not a big problem | 1.26(1.04-1.51)** | 0.87(0.69-1.09) | 1.68(1.32-2.13) | 0.93(0.67-1.28) | 1.50(1.13-2.01)** | 1.11(0.76-1.61) | 0.80(0.62-1.04)* | 0.93(0.69-1.25) |
| **Covered by Health Insurance** |  |  |  |  |  |  |  |  |
| No | Reference(1.0) | Reference(1.0) | Reference(1.0) | Reference(1.0) | Reference(1.0) | Reference(1.0) | Reference(1.0) | Reference(1.0) |
| Yes | 1.53(0.94-2.49)* | 0.81(0.48-1.36) | 5.25(2.84-9.71)*** | 1.62(0.85-3.08) | 2.11(0.68-6.66) | 0.83(0.22-3.12) | 1.04(0.43-2.52) | 0.82(0.30-2.31) |
| **Husband level of education** |  |  |  |  |  |  |  |  |
| None | Reference(1.0) | Reference(1.0) | Reference(1.0) | Reference(1.0) | Reference(1.0) | Reference(1.0) | Reference(1.0) | Reference(1.0) |
| Primary | 2.35(1.74-3.18)*** | 1.44(1.02-2.02)** | 1.86(1.37-2.52)*** | 1.38(0.97-1.96)* | 0.93(0.54-1.62) | 0.86(0.50-1.47) | 0.82(0.48-1.40) | 0.78(0.45-1.33) |
| Secondary | 3.54(2.72-4.62)*** | 1.49(1.06-2.09)** | 3.00(2.21-4.08)*** | 1.58(1.09-2.29)** | 1.45(1.04-2.01)** | 1.25(0.87-1.79) | 0.68(0.39-1.17) | 0.70(0.40-1.23) |
| Higher | 3.69(2.74-4.98)*** | 1.30(0.87-1.94) | 8.39(4.19-16.80)*** | 2.81(1.31-6.02)** | 2.79(1.76-4.42)*** | 1.97(1.18-3.27)** | 0.92(0.48-1.77) | 0.91(0.41-2.01) |
| **Difference in age between husband and wife** |  |  |  |  |  |  |  |  |
| Wife older or same age | Reference(1.0) | Reference(1.0) | Reference(1.0) | Reference(1.0) | Reference(1.0) | Reference(1.0) | Reference(1.0) | Reference(1.0) |
| Husband 1-5 years older | 1.40(0.76-2.58) | 1.35(0.70-2.60) | 0.46(0.22-0.94)** | 0.45(0.21-0.97)** | 0.98(0.36-2.71) | 1.00(0.32-3.10) | 1.89(0.97-3.68)* | 1.73(0.95-3.16)* |
| Husband 6-10 years older | 1.07(0.59-1.93) | 1.35(0.72-2.56) | 0.52(0.26-1.05)** | 0.54(0.25-1.16) | 1.01(0.38-2.67) | 1.00(0.32-3.08) | 1.65(0.84-3.23) | 1.62(0.88-3.00) |
| Husband > 10 years older | 0.95(0.53-1.71) | 1.52(0.80-2.88) | 0.60(0.30-1.19) | 0.56(0.27-1.19) | 1.23(0.47-3.24) | 1.34(0.44-4.05) | 2.43(1.15-5.10)** | 2.43(1.24-4.78)** |

***p<0.001,**p<0.05,*p<0.10
